# Altered Expression of an *FT* Cluster Underlies a Major Locus Controlling Domestication-Related Changes to Chickpea Phenology and Growth Habit

**DOI:** 10.3389/fpls.2019.00824

**Published:** 2019-07-03

**Authors:** Raul Ortega, Valerie F. G. Hecht, Jules S. Freeman, Josefa Rubio, Noelia Carrasquilla-Garcia, Reyazul Rouf Mir, R. Varma Penmetsa, Douglas R. Cook, Teresa Millan, James L. Weller

**Affiliations:** ^1^School of Natural Sciences, University of Tasmania, Hobart, TAS, Australia; ^2^Scion, Rotorua, New Zealand; ^3^E. Genomica y Biotecnologia, Instituto Andaluz de Investigación y Formación Agraria y Pesquera (IFAPA), Córdoba, Spain; ^4^Department of Plant Pathology, University of California, Davis, Davis, CA, United States; ^5^Division of Genetics and Plant Breeding, Sher-e-Kashmir University of Agricultural Sciences and Technology of Kashmir, Srinagar, India; ^6^Department of Plant Sciences, University of California, Davis, Davis, CA, United States; ^7^Department of Genetics ETSIAM, University of Córdoba, Córdoba, Spain

**Keywords:** chickpea, domestication, florigen, flowering, growth habit, legume, photoperiod, QTL

## Abstract

Flowering time is a key trait in breeding and crop evolution, due to its importance for adaptation to different environments and for yield. In the particular case of chickpea, selection for early phenology was essential for the successful transition of this species from a winter to a summer crop. Here, we used genetic and expression analyses in two different inbred populations to examine the genetic control of domestication-related differences in flowering time and growth habit between domesticated chickpea and its wild progenitor *Cicer reticulatum*. A single major quantitative trait locus for flowering time under short-day conditions [*Days To Flower* (*DTF)3A*] was mapped to a 59-gene interval on chromosome three containing a cluster of three *FT* genes, which collectively showed upregulated expression in domesticated relative to wild parent lines. An equally strong association with growth habit suggests a pleiotropic effect of the region on both traits. These results indicate the likely molecular explanation for the characteristic early flowering of domesticated chickpea, and the previously described growth habit locus *Hg*. More generally, they point to de-repression of this specific gene cluster as a conserved mechanism for achieving adaptive early phenology in temperate legumes.

## Introduction

The timing of flowering is a critical trait for crop adaptation, and as such has significant implications for yield and economic output (Jung and Muller, 2009; Nelson et al., 2010). The wild forms of many crops have a strong environmental requirements for flowering, ensuring that seed development occurs under favorable conditions. However, such requirements often constitute a physiological barrier for adaptation to wider agro-ecological ranges, and in general, domestication and subsequent diversification has involved selection of variants in which these requirements have been modified. A well-known example is wheat (*Triticum aestivum* L.), where relaxation of photoperiod and vernalization responses has allowed the development of spring cultivars (Trevaskis et al., 2003; Yan et al., 2003; Fu et al., 2005; Beales et al., 2007; Díaz et al., 2012; Kippes et al., 2015, 2016). Similar adaptations have been reported in many other species (Nakamichi, 2015), including legumes, where a loss-of-function mutation in the circadian clock gene *ELF3* overcame the obligate LD requirement of pea (*Pisum sativum* L.), permitting its conversion from a winter to a spring crop at higher latitudes (Weller et al., 2012). Similarly, a mutation at the *Ppd* locus in the short-day species common bean (*Phaseolus vulgaris* L.) enabled summer cropping and broad global adaptation of this crop (Wallace et al., 1993; Weller et al., 2019).

Chickpea (*Cicer arietinum* L.) is a major grain legume, ranking third in global production after bean and pea (FAO, 2016). It is more drought-tolerant than other cool season legumes, and its relative importance is projected to increase in future due to global population growth and climate change (Bar-El Dadon et al., 2017; Muehlbauer and Sarker, 2017). Despite being domesticated in parallel with other long day vernalization-responsive legumes (pea, lentil) and cereals (wheat, barley) (Zohary and Hopf, 2000), the domestication history of chickpea is distinct from these other species (Abbo et al., 2003a). One key difference is the decline of chickpea in the archeological record between the Neolithic period, approximately 9000 years before present (ybp) and the early Bronze Age (approximately 5000 ybp) (Abbo et al., 2003b). A second key difference is that across its center of origin, chickpea has traditionally been grown as a summer crop (Abbo et al., 2003b), and varieties with the winter annual habit typical of wild chickpea are notably absent. This contrasts with other species domesticated in the Fertile Crescent region over the same period, such as barley and pea, in which a significant proportion of the domesticated germplasm retains the ancestral, wild phenology (Saisho et al., 2011; Weller et al., 2012).

The reasons for these two differences are not known, but it is thought that chickpea was neglected as a winter crop in favor of other pulses, as a result of its inherently greater susceptibility to Ascochyta blight, a fungal disease caused by *Ascochyta rabiei*. This disease can cause total crop failure, particularly during humid Mediterranean winter conditions (Siddique et al., 2000; Millan et al., 2003; Sharma and Ghosh, 2016) and its impact would likely have intensified as planting densities increased with cultivation. This pressure may have motivated attempts by early farmers to shift cultivation from autumn sown, over-winter crop (when most precipitation occurs in this region) to a spring-sown summer crop that matures in the predominantly dryer summer season. In such a scenario the selection of earlier-flowering genotypes able to complete their life cycle prior to the onset of summer drought would likely have been essential (Kumar and Abbo, 2001), and the increase in the frequency of archaeobotanical remains of chickpea in the Bronze Age is suggested to reflect the success of this transition (Kumar and Abbo, 2001; Abbo et al., 2003a).

Early phenology continues to be important in present-day chickpea cultivation, as a large proportion of the global chickpea crop is grown in short season environments exposed to end-of season stresses that reduce their productivity (Kumar and Abbo, 2001; Muehlbauer and Sarker, 2017). In Mediterranean and semi-arid environments, where chickpea is grown under rain-fed conditions and matures into summer, terminal drought is the most common cause of yield loss (Zhang et al., 2000; Turner et al., 2001; Siddique et al., 2003; Berger and Turner, 2007). In higher-latitude continental temperate environments like western Canada, the short growing season is instead limited by declining temperatures, delayed maturity and increased potential for frost damage at the sensitive phase of pod development (Croser et al., 2003; Berger J.D. et al., 2004; Clarke and Siddique, 2004; Anbessa et al., 2007). In both situations, early flowering and maturity is thus an important primary escape strategy (Siddique et al., 2003; Berger J.D. et al., 2004, 2006) Hence, genetic control of this trait has been a topic of increasing interest (e.g., Gaur et al., 2008; Ridge et al., 2017).

Several flowering time loci have been reported in chickpea from both classical and quantitative trait locus (QTL) analyses. These include four major loci; *Photoperiod* (Or et al., 1999), *Early flowering 1* (*Efl1*), *Efl3*, and *Efl4* (Kumar and Van Rheenen, 2000; Hegde, 2010; Gaur et al., 2014), and several QTL that appear recurrent in different populations. One prominent example is a “hot-spot” on linkage group (LG) four (Cobos et al., 2007; Varshney et al., 2014; Daba et al., 2016; Mallikarjuna et al., 2017). Another important genomic region is the central portion of chromosome 3 between markers TA6 and TA64, in which flowering time QTL have been reported from all wide crosses investigated for this trait (Cobos et al., 2009; Aryamanesh et al., 2010; Das et al., 2015; Samineni et al., 2015), as well as in several other intraspecific populations (Hossain et al., 2010; Hamwieh et al., 2013; Daba et al., 2016; Mallikarjuna et al., 2017).

In this study we aimed to elucidate the genetic basis of changes in flowering time that occurred early in chickpea crop evolution, through QTL analysis and candidate gene evaluation in two recombinant inbred populations between *Cicer arietinum* and its wild progenitor *C. reticulatum*. Our results point to a strong genetic association between the early flowering and erect growth habit typical of domesticated chickpea, and the elevated expression of a cluster of *FT* genes on chromosome 3. We conclude that a *cis*-acting genetic change leading to deregulated expression of this gene cluster may have played a key role in the prehistoric shift in phenology and farming practice integral to chickpea evolution under domestication.

## Materials and Methods

### Plant Material

CRIL2 is a recombinant inbred line (RIL) population developed from an interspecific cross between *C. arietinum* (accession ICC4958) and *C. reticulatum* (PI489777) by Tekeoglu et al. (2000), Winter et al. (2000), Muehlbauer and Sarker (2017) at the United States Department of Agriculture (USDA), Agricultural Research Service and Washington State University, United States. ICC4958 is an early-flowering desi chickpea type with an erect growth habit, while the wild parent PI489777 is a Turkish accession with prostrate growth habit and late flowering typical of wild chickpea.

Three other recombinant inbred populations were used in this study, developed by the chickpea breeding group in IFAPA (Institute of Agricultural and Fisheries Research and Training, Centro Alameda del Obispo, Cordoba, Spain) and University of Córdoba, Spain. RIP12 is an interspecific population consisting of 88 F_6:7_ RILs derived from a cross between the kabuli cultivar ICCL81001 and a *C. reticulatum* accession, as described in Cobos et al. (2009). RIP5 (102 RILs) and RIP8 (113 RILs) are two F_6:8_ RIL populations derived from reciprocal crosses between the early flowering desi landrace WR315 and the late kabuli accession ILC3279 (Iruela et al., 2007; Ali et al., 2015).

### Growing Conditions and Phenotypic Evaluation

Four plants of each of the CRIL2 parents and 124 RILs were grown under long day (LD) or short day (SD) conditions in an automated phytotron at the University of Tasmania between December 2015 and April 2016. Plants under SD received 8 h (8 AM–4 PM) of natural daylight and were then moved to complete darkness inside the phytotron. Plants under LD received natural daylight, extended throughout the growing season with artificial light from high-pressure sodium lamps (50 μmolm^–2^ s^–1^) to provide a total photoperiod of 18 h. Night temperature inside the phytotron was maintained at 16°C. Flowering time was recorded as the number of days from seedling emergence to opening of the first flower (DTF) on each individual plant. Lines remaining vegetative at 130 days were assigned a nominal DTF value of 130 in subsequent analyses. Branching tendency was quantified at 3 weeks after emergence and expressed as the ratio of total branch length to main shoot length (branching index, BI) to normalize for differences in general vigor and stem elongation. Growth habit (GH) was scored using a four-category scale (values from 1 to 4), according to the angle of the branches from the vertical axis at harvest stage, as follows: (1) prostrate (branches 0–10° above horizontal), (2) semi-prostrate (10–45°), (3) semi-erect (45–70°), and (4) erect (>70°). For all three traits, the mean value from the four replicate plants was used for analysis.

RIP12 was sown in March in the field at the IFAPA site in Cordoba (latitude/longitude/altitude: 37°53′N/4°47′W/117 m) over four different seasons (2001, 2004, 2008, and 2014). Plots consisted of 2 m-long rows set 0.5 m apart, each sown with 20 plants of each RIL. Every fifth row was sown with one of the parent lines as a check. In 2001, a greenhouse trial was also conducted to assess flowering time under natural short day conditions (Cobos et al., 2009). RIP5 was sown in the field in March 2003 at two different sites: the IFAPA site in Cordoba and the IFAPA Venta del Llano site (Mengibar, Jaen, Spain; latitude/longitude/altitude: 37°57′N/3°48′W/280 m). In this trial, RILs were randomly distributed in four blocks and parents were included as reference in each trial. The unit plot was two rows of 2 m, with 10 seeds/m and 0.7 m between rows (Ali et al., 2015). RIP8 was sown in the field in February 2003 at the IFAPA site in Cordoba with two replications, in which RILs were distributed randomly into four blocks with 20 lines per block. Four check lines were included in each block following a Latin square design to verify environmental homogeneity. The plot unit was three rows, 4 m long, with 0.5 m between rows and a density of 20 plants m^–2^. For these three populations, days from sowing to 50% flower was recorded (DTF). The data obtained from each of the two trials of RIP8 were analyzed separately. Information about the photoperiod experienced by RIP12, RIP5, and RIP8 during the different growing seasons can be found in Supplementary Table 5.

### Molecular Markers

Both markers from previous linkage maps and new markers developed specifically for this study were used for map construction and QTL analysis. Polymorphisms in target genes across chickpea LG3 and LG4 were identified by sequencing of the parental accessions or from information available in previous reports (Saxena et al., 2014), and used to design 27 high-resolution melt (HRM) markers (Supplementary Table 1) that were added to the markers previously genotyped in the RIP12, RIP5, and RIP8 populations previously described in Iruela et al. (2007), Cobos et al. (2009), Ali et al. (2015), respectively. In the case of CRIL2, the HRM markers were combined with a subset of 210 molecular markers selected from a dense map incorporating 2956 markers (Supplementary Figure 1; von Wettberg et al., 2018), to provide an even distribution [approximately 1 marker/5 centiMorgan (cM)] of high-quality (minimal missing data) markers (Supplementary Table 1).

### Genetic Mapping and QTL Analysis

Linkage analysis in each population was performed using JoinMap v4.0 (Van Ooijen, 2006). Markers were grouped with a minimum logarithm of odds (LOD) value of 3.0, and the regression algorithm was used for mapping, using default options and the Kosambi function for the estimation of genetic distances (Kosambi, 1943). The initial maps were reviewed and problematic markers were removed where necessary based on the following criteria: Chi-square goodness-of-fit threshold (>1); nearest neighbor fit; genotype probability function; and the level of segregation distortion compared to surrounding markers. Following the removal of problematic markers, the maps were re-calculated and the process repeated where necessary, until maps with robust order were produced.

The numbering of the LGs followed the chickpea consensus genetic map (Millan et al., 2010), based on the presence of markers in common with the consensus map itself or others marker of known position, using the Cool Season Food Legume Database^1^.

Quantitative trait locus analysis was performed using MapQTL6.0 software (Van Ooijen, 2009). First, interval mapping was carried out to detect putative QTL associated with the variation in each trait. For each putative QTL, the marker closest to the LOD peak and two markers either side of this were used in Automatic Cofactor Selection (ACS) to select the best cofactor for subsequent Multiple QTL Mapping (MQM) analysis. The MQM function was employed iteratively with each new cofactor selection until all QTLs for a specific trait were determined. In both interval and MQM mapping, putative QTL were declared at a chromosome-wide threshold (*p* < 0.05) based on permutation testing with 1000 permutations.

### RNA Extraction and qPCR

For the expression study, the six parental lines of the four populations (RIP5 and RIP8 share the same parental accessions, and therefore were represented only once) were grown in an automated phytotron at the University of Tasmania under SD (8 h) and LD (16 h) conditions. For quantitative reverse-transcriptase PCR (qRT-PCR), dissected apical buds and the uppermost fully expanded leaflets were harvested. Each sample consisted of pooled material from two plants, harvested at midday at 2–4 weeks after seedling emergence. RNA extraction, cDNA synthesis and gene expression determination were performed as described in Sussmilch et al. (2015) using the primers indicated in Supplementary Table 2. The expression level of tested genes was normalized against *ACTIN* using the ΔΔCt method.

### Statistical Analysis

Statistical analysis was conducted using IBM SPSS Statistics (version 22), including box-plot and frequency distribution graphs. Correlation between traits was measured using Spearman’s rank correlation coefficient, and statistical significance was tested by paired or independent *t*-test, according to the nature of the data.

## Results

### A Major Locus Controls Flowering in the CRIL2 Interspecific Reference Population

We initially characterized flowering time in the CRIL2 reference population under controlled 8-h SD and 18-h LD conditions in an automated phytotron. Phenotypic values obtained are summarized in Supplementary Figure 2. Under LD, the difference in flowering time between the parental lines was not significant, with both flowering between 30 and 33 days after emergence. In contrast, under SD, ICC4958 flowered at around 60 days while PI489777 remained vegetative until the experiment was terminated 130 days after sowing. Thus, under these conditions, ICC4958 shows a moderate, quantitative response to photoperiod, whereas the wild line shows an obligate requirement for LD.

Among the RILs, the mean DTF under LD conditions was intermediate between the two parents while the range was substantially wider, with 12 days difference between the minimum and maximum values. Under SD, flowering time in the CRIL2 population showed a clear bimodal distribution, with a significant proportion of lines (68 out of 124) failing to initiate flowering by 130 days after sowing, like the wild parent. All RILs flowered considerably later under SD than under LD (*p* < 0.001) but, interestingly, phenotypic values for DTF in the two conditions were significantly correlated (with only 56 RILs able to flower in both LD and SD considered; *rs*[56] = 0.500, *p* < 0.001), indicating that part of the variation is independent of photoperiod. Transgressive segregation, particularly toward earliness, was observed under both photoperiods (Supplementary Figure 2), suggesting that alleles associated with early flowering have been contributed from both parents.

**FIGURE 1:**
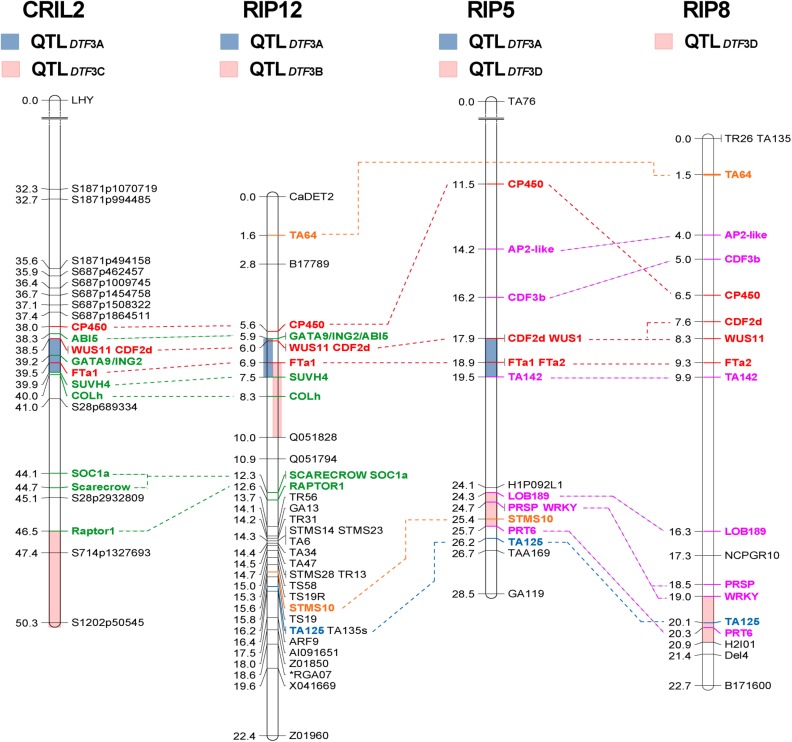
Comparative mapping of flowering time QTLs on chromosome 3. Four regions of chromosome 3 (3A to 3D, colored bars) were found to influence flowering time across three different populations. The length of the bars representing each QTL indicates the two-LOD support interval, which corresponds to a ∼95% confidence interval (Van Ooijen, 2006). Only region 3A, in the central portion of the chromosome and containing a cluster of *FT* genes, is consistently detected in both narrow and wide crosses. Numbers at the left of the bars represent genetic distance (in cM). Common markers were used to compare the relative position of the QTLs across populations. Markers common to all populations are shown in red, to three populations in blue and to two populations in orange. Those common to both interspecific populations are shown in green, and to both intraspecific populations, in pink.

Consistent with the phenotypic homogeneity observed for flowering time in CRIL2 under LD, QTL analysis under these conditions revealed only one minor QTL, *DTF3C* (Table 1), located at the top of LG3 (Figure 1). In contrast, under SD conditions, a major effect QTL, *DTF3A*, was found in the middle of LG3 (LOD 50.2, PVE 85). As the peak markers for these loci are separated by only around 10 cM, and the effective population size for the LD analysis is relatively small, the possibility that the loci may be the same cannot be excluded. However, as it is also not trivial to prove, we have adopted a conservative interpretation and assigned them distinct names.

**TABLE 1:** Quantitative trait loci (QTL) identified by multiple QTL mapping for flowering time, growth habit and branching index in four populations grown in different environments.

**Population**	**Place**	**Year**	**Trait^a^**	**Cond^b^**	**QTL**	**LOD^c^**	**PVE^d^**	**Marker^e^**	**LG^f^**	**Early^g^**	**Late^g^**	**Thr^h^**
**CRIL2**	Hobart	2016	DTF	LD, P	*DTF*3C	2.9	9.6	S1202p50545	3	29	30.3	2.8
				SD, P	*DTF*3A	50.2	85.2	*FT*a1	3	66.2	128.6	2.6
		
		2016	GH	SD, P	*GH*3	34	66.6	*FT*a1	3	3.4	1.6	2.6
					*GH*4	5.5	5.9	S360p1277380	4	2.8	2.2	3.1
		
		2016	BI	SD, P	*BI*3	10.6	33.1	*FT*a1	3	0.4	0.9	2.6
				LD, P	*BI*3	5.4	18.4	*FT*a1	3	0.2	0.5	2.5

**RIP12**	Cordoba	2001	DTF	GLH	*DTF*3A	10.8	46.9	*FT*a1	3	14.1	39.6	3.1
				Field	*DTF*3A	4.5	22	*FT*a1	3	60.4	68.6	2.9
		
		2004	DTF	Field	*DTF*3A	14.8	51.1	*FT*a1	3	8.9^i^	21.7^i^	2.9
					*DTF*4B	3.6	9.2	STMS11	4	17.9^i^	12.6^i^	3.3
		
		2008	DTF	Field	*DTF*3B	6.3	29.6	COLh	3	70.3	76.8	2.9
		
		2014	DTF	Field	*DTF*3A	8.4	29.8	*FT*a1	3	58.3	64.2	3
					*DTF*4A	5.3	17.3	GAA47	4	63.5	59	2.8

**RIP5**	Cordoba	2003	DTF	Field	*DTF3D*	9.6	38.7	WRKY	3	60.4	64.8	2.7
	Cordoba				*DTF*3A	3	8.7	*FT*a1/2	3	61.3	63.9	2.7
	Mengibar				*DTF*3A	5.7	26.9	*FT*a1/2	3	64.2	66.7	2.8

**RIP8**	Rep1	2003	DTF	Field	DTF3D	7.5	29.2	TA125	3	84.3	87.3	2.6
	Rep2				DTF3D	6.8	29.0	TA125	3	84.6	87.3	2.7

*^*a*^Trait analyzed: DTF, flowering time; GH, growth habit; BI, branching index. ^*b*^Condition: LD, long days; SD, short days; P, phytotron; GLH, glasshouse. ^*c*^The LOD scores for each QTL. ^*d*^PVE, Phenotypic variation explained. ^*e*^Marker nearest to the peak LOD score. ^*f*^LG, linkage group harboring the QTL. ^*g*^Marker genotype class means for early (*C. arietinum* accessions ICC4958, ICCL81001 and WR315 for CRIL2, RIP12, and RIP5/8, respectively) and late (*C. reticulatum* accessions PI489777 and Cr5-9 in CRIL2 and RIP12 and *C. arietinum* ILC3279 in the case of RIP5/8) parents, calculated for the marker with higher LOD. ^*h*^Threshold LOD for a 0.995 confidence value, calculated through permutation test for each trait and linkage group. ^*i*^Flowering time in 2004 is a relative value, as specified in Supplementary Figure 2.*

Quantitative trait locus analysis was also performed using a subset of the population formed by those 56 RILs that were able to flower under both SD and LD. Interestingly, no significant QTL were found in this case, supporting the idea that only QTL *DTF3A* is acting in CRIL2 grown under SD. However, these results should be interpreted with caution, considering the small population size.

### Mapping Identifies the *FT* Cluster as Strong Positional Candidates for *DTF*3A

Several previous studies have reported major flowering QTLs in the central region of chromosome 3 between markers TA6 and TA64 (summarized in Supplementary Figure 3), indicating this as a particularly important genomic region (Weller and Ortega, 2015). We scanned this region for genes similar to known flowering time genes in other species and added 18 additional markers to the CRIL2 linkage map, including 13 within the TA6-TA64 interval (Supplementary Figure 3 and Supplementary Table 1). This confirmed the presence of *DTF3A* within this interval and narrowed its location to a smaller interval flanked by markers SUVH4 and CDF2d (Figure 1), that corresponds to a physical distance of 1.4 Mbp and contains 124 annotated genes, according to the reference genome. Many of the flowering-related genes annotated in this region lie outside of this interval and were thus considered to be unlikely candidates, including *SOC1a* (*SUPPRESSOR OF CONSTANS OVEREXPRESSION 1*), *COLh* (*CONSTANS-LIKE h*), *AG* (*AGAMOUS*)*-like*, *LUX* (*LUX ARRHYTHMO*)*-like*, *CDF* (*CYCLING DOF FACTOR*), and *WRKY* (Supplementary Figure 3). However, the analysis confirmed the presence of a cluster of *FT* genes directly under the QTL peak, and a marker for one of these, *FTa1*, showed the strongest association with SD flowering time among all the markers tested (Table 1).

**FIGURE 2:**
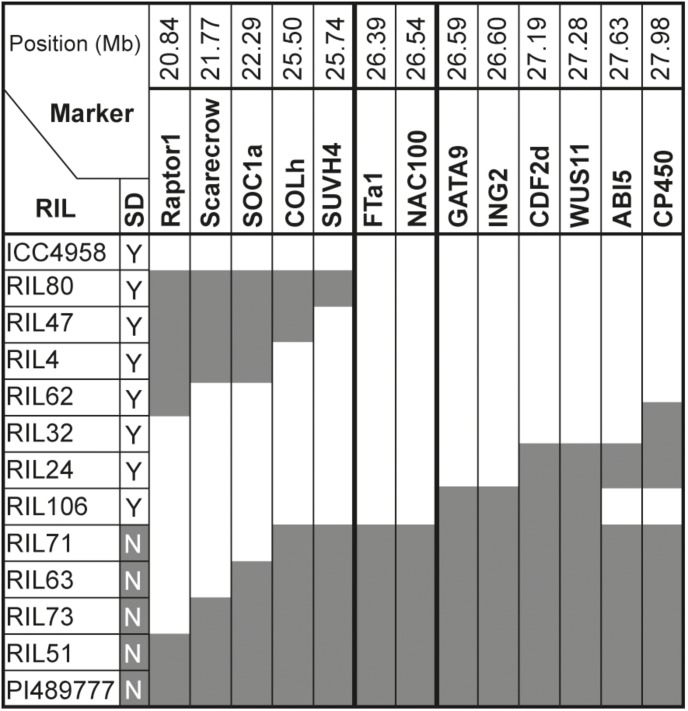
Refinement of the *DTF3A* location. Marker genotypes in recombinant inbred lines from the CRIL2 population showing recombination breakpoints across a 7.14 Mb region of chromosome 3 spanning the *DTF3A* locus. Numbers over the markers correspond to their physical position (in Mb) in the CDC Frontier genome assembly in NCBI (ASM33114v1; Varshney et al., 2013). Alleles from the domesticated parent ICC4958 are shown in white and those from the wild parent PI489777 in gray. Flowering phenotype is shown in the column headed SD and indicates whether the indicated lines flowered (Y) or remained vegetative (N) under an 8h photoperiod. This phenotype showed no recombination between markers *FTa1* and GATA9.

The dramatic delay in flowering of the PI489777 parent line and the bimodal distribution of the flowering phenotypes in CRIL2 under SD suggested that the QTL could also be analyzed as a single Mendelian locus, to refine its position. Figure 2 illustrates all recombinants identified in the CRIL2 population across the LG3A region, and shows that *DTF3A* can be further delimited to a region of 0.8 Mb between markers *SUVH4* and *GATA9*/*ING2* (Supplementary Table 3). This region contains only 59 genes, but still includes the *FT* cluster.

### Comparison of the DTF3A Region in Other Crosses

The segregation of a major flowering time locus in CRIL2 and several other interspecific populations suggests a potential role for this locus in early crop evolution. However, a lack of common markers has made it difficult to compare the position of QTL between studies and clearly demonstrate their co-location. To investigate the position of *DTF3A* relative to previously described QTLs, and assess the possible relevance of this region at the intraspecific level, we selected three additional populations for parallel analysis through mapping of common markers. RIP12 is another interspecific population, for which a major flowering QTL has been reported in the TA6-TA64 region (Cobos et al., 2009). The intraspecific populations RIP5 and RIP8 were also examined, as preliminary evidence indicated an association of markers in the 3A region with flowering time in this cross (Castro, 2011). Where polymorphisms were available, the genes targeted in CRIL2 were also genotyped and added to the linkage maps in these additional populations (Supplementary Table 1) by recalculation of the linkage maps with markers for these genes and previously mapped markers (Supplementary Figures 4–7). These maps were then used for QTL analysis of flowering data for the three populations across different locations, years, and environments (Supplementary Figure 2), revealing a total of 12 significant flowering QTL (Table 1).

In the RIP12 population, analysis over several years, in glasshouse and field environments, yielded seven QTL; five on LG3 and two on LG4 (Table 1). The QTL on LG3 were defined by the same interval 3A described above for CRIL2 (Figure 1), and the *FTa1* marker again explained the highest proportion of variation (up to 51%). During 2008, a flowering QTL *DTF3B* was detected in a second region of LG3 between markers *FT*a1 and Q05_182__8_. Since both the position of the interval (Figure 1) and the significance of the QTL (∼30% PVE) are very close to those obtained for *DTF3A* (Table 1), it seems highly probable that these two QTL are equivalent.

In the intraspecific populations, two regions on chromosome 3 influenced flowering time. One of these was region *3A*, which was detected in the RIP8 population, with a variable effect on flowering time depending on location, with a strong effect when grown in Mengibar, and a weaker influence in Cordoba (26.9 vs. 8.7% variance explained, respectively). An additional highly significant QTL (*DTF3D*) was detected on LG3, between markers LOB189 and PRT6, in both intraspecific populations (Figure 1). Although this QTL was not detected in RIP5 at Mengibar, in situations where it was detected it had a greater effect than *DTF3A* (Table 1).

### *FT* Genes in Chickpea

In view of the central location of an *FT* gene cluster under the *DTF3A* QTL, we characterized the entire chickpea phosphatidylethanolamine-binding protein (PEBP) family, which includes *FT* genes and the related *TFL1* (*TERMINAL FLOWER 1*) family of flowering repressors (Wickland and Hanzawa, 2015; Supplementary Figures 8A,9 and Supplementary Table 4). Five chickpea *FT*-like genes were identified in the three previously described legume *FT* subclades; *FTa*, *FTb*, and *FTc* (Supplementary Figure 10; Hecht et al., 2011). This analysis confirmed that chickpea, like Medicago, possesses three *FTa* genes, with two of these (*FTa1* and *FTa2*) located together with the single *FTc* gene on chromosome 3 in a tandem arrangement (Hecht et al., 2011; Laurie et al., 2011). Only one other PEBP gene was found on this chromosome (*TFL1a*), while the remaining genes were located on chromosomes 1 (*TFL1b*), 2 (*FTb* and *FTa3*), 6 (*MOTHER OF FT*, *MFT*), and 8 (*TFL1c*) (Supplementary Figure 8B). The only difference in the chickpea *FT* family compared to other related legume species is the apparent presence of only a single *FTb* gene, where Medicago and pea each have two highly similar paralogs located in tandem in a conserved genomic location on chromosome 7 and LG5, respectively (Hecht et al., 2011; Laurie et al., 2011). In the broader PEBP family, chickpea possesses single-copy orthologs of the *BFT* (*BROTHER OF FT*) and *MFT* genes, and also of two of the three *TFL1* genes previously described in pea and Medicago, *TFL1a* and *TFL1b*. The third gene, *TFL1c*, was represented by three gene models in the CDC Frontier genome assembly (Supplementary Table 4), but was not represented at all in the other available chickpea genome (from ICC4958, assembly ASM34727v3); a discrepancy that will require clarification in future.

### Genes in the *FT*a1-*FT*a2-*FT*c Cluster Are Upregulated in Early Accessions

*FT* genes are well-known as important positive regulators of flowering. This is also true in legumes, where several *FT* genes have been identified and most are capable of promoting flowering when overexpressed in Arabidopsis (Kong et al., 2010; Hecht et al., 2011; Laurie et al., 2011; Sun et al., 2011). Therefore, if one of the *FT* genes in the cluster was the basis for the effect of the *DTF3A* locus, increased activity or expression of one or more of these genes would be expected in the early-flowering parent. To evaluate this possibility, we examined the expression of *FT* genes in the parent lines of the mapping populations. In view of previous reports indicating tissue- and photoperiod-specific expression of *FT* genes in pea and Medicago, we collected samples from leaf and apex tissue under both LD and SD conditions at two timepoints. Expression of the *AP1* homolog *PROLIFERATING INFLORESCENCE MERISTEM* (*PIM*) was used as an indicator of flowering commitment, as previously described for other legumes including chickpea (Hecht et al., 2011; Ridge et al., 2016).

**FIGURE 3:**
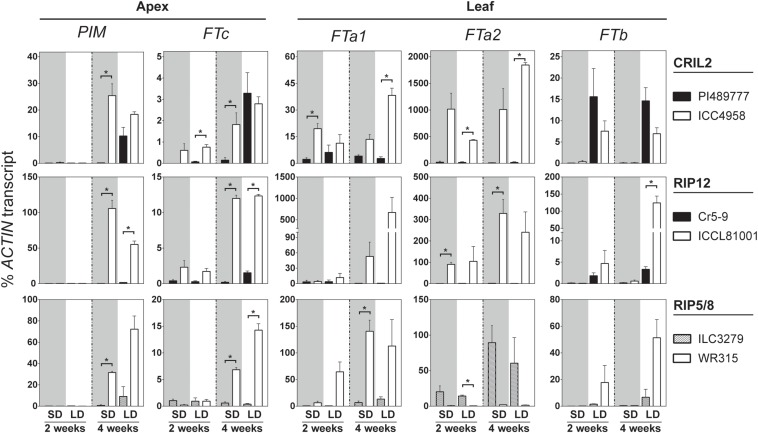
*FT* genes in the LG3 cluster are upregulated in the early parents of the crosses. Relative expression profiles of *FT* genes and the floral indicator *PIM* in the parental lines of the four chickpea populations analyzed. Expression was measured in dissected apical bud or leaves of plants grown from sowing under short (SD, gray background) and long days (LD, white background) for 2–4 weeks. Late flowering lines are shown in black and early parents in white. The average ± SE of two biological replicates (two technical replicates each) is shown, and transcripts were normalized against *ACTIN*. Asterisk indicates significant difference (*p* < 0.05) in the level of expression (*t*-test).

Figure 3 shows that 2 weeks after emergence *PIM* expression in shoot apices was not detectable in any of the accessions. By 4 weeks, *PIM* was expressed significantly above background in all three late parents under LD but not in SD, whereas it was strongly expressed under both LD and SD in the early parents. In parallel, the expression of all three genes in the chromosome 3 *FT* cluster (*FTa1*, *FTa2*, and *FTc*) was elevated in the early parents at 4 weeks under SD and LD. In ICC4958, expression of all three genes was higher than the wild parent even by week 2; i.e., before detectable expression of *PIM*. Similarly, expression of *FTa2* and *FTc* was also elevated in the early parent of RIP12 (ICCL81001) at week 2. However, *FTa2* transcript could not be detected in the early parent of RIP5/8 (WR315), reflecting a complete deletion of the gene (Supplementary Figure 11). This result suggests that the elevated expression of *FTa2* seen in the domesticated parents of CRIL2 and RIP12 is unlikely to be solely responsible for the effect of *DTF3A* in these populations. As in pea and Medicago, *FTa1* and *FTc* in chickpea differed in the tissue-specificity of their expression, with *FTa1* expressed strongly in leaves and weakly at the shoot apex, and *FTc* expressed only weakly at the shoot apex. Despite these differences, both genes showed similar expression profiles, with an early upregulation in the domesticated/early flowering parents that preceded *PIM* induction, and they therefore represent good candidates to underlie the QTL.

Significant expression of the single *FTb* gene was seen in 2-week-old plants, but only under LD, and at a similar level in both early and late parents. This is similar to the strongly photoperiod-dependent expression of *FTb* genes previously reported in pea and Medicago (Hecht et al., 2011; Laurie et al., 2011), and indicates that *FTb* misexpression is not a factor in the effect of *DTF3A* under SD. The expression of *FTa3* was restricted to leaf tissue, and only detected at a late developmental phase after commencement of flowering (Supplementary Figure 12), suggesting it is unlikely to make a major contribution to the observed differences in flowering time. The expression of *TFL1b* and *TFL1c* was also tested in apical tissue. Whereas expression of *TFL1c* in this tissue did not change significantly, *TFL1b* expression was higher in the wild line under non-inductive conditions and gradually decreased in cultivated and wild accession grown in long photoperiod, consistent with a possible role as a floral repressor. However, the level of expression observed in both genes was very low and the biological significance of these changes is therefore uncertain (Supplementary Figure 12).

### The DTF3A Locus Coincides With QTL for Plant Architecture

The late-flowering phenotype of wild chickpea is also associated with a prostrate growth habit (GH), reduced apical dominance and an increased number of branches (Singh and Shyam, 1959; Aryamanesh et al., 2010; Ali et al., 2015). Consistent with these reports, we also observed major differences in growth habit between CRIL2 parents and in the CRIL2 population in SD, which we quantified for genetic analysis using a four step scale (Supplementary Figures 13A–D). We also recorded branching propensity in young plants (prior to visible flower initiation) under both SD and LD. Late flowering RILs also showed a shoot architecture that resembled the wild parent, so we investigated the correlation between these three traits (Supplementary Figure 13). A highly significant difference (*p* < 0.001) was found between the flowering dates of erect/semierect RILs compared to those with a prostrate/semiprostrate growth habit (Supplementary Figure 13E), confirming that in the segregating population, prostrate growth habit is associated with late flowering, as expected. Inspection of individual RILs showed a nearly perfect correlation, with flowering observed in all 53 erect or semi-erect RILs but in only three out of 71 lines categorized as prostrate or semi-prostrate. A strong negative correlation (*r* = −0.504, *p* < 0.001) was found between growth habit and branching index (Supplementary Figure 13F), indicating that erect and semi-erect plants in general also had a lower branching index (BI).

**FIGURE 4:**
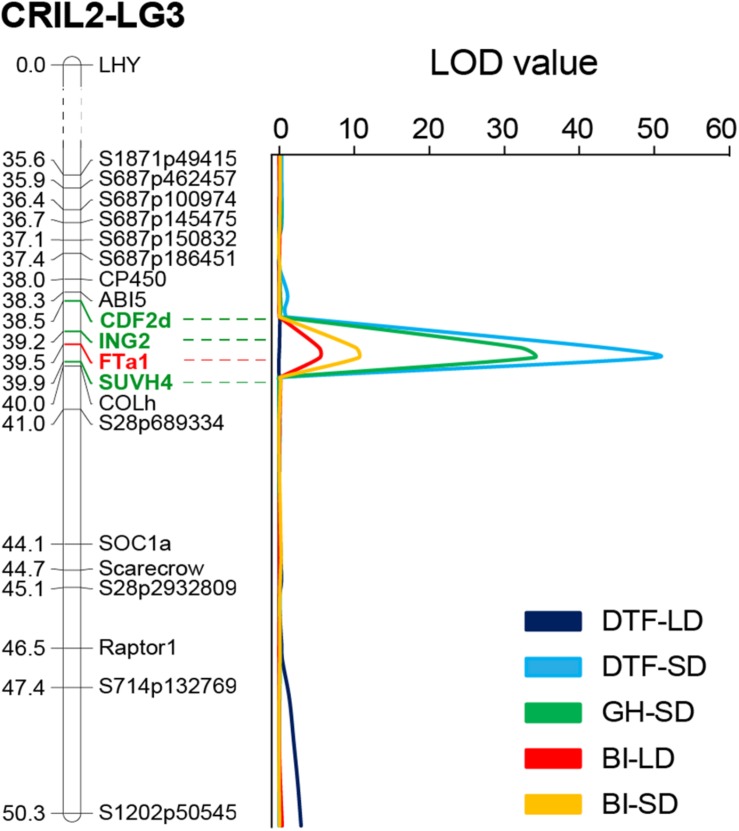
The DTF3A region is also associated with growth habit and shoot branching. Portion of the Linkage Group three from the CRIL2 genetic map showing the perfect co-location of QTLs obtained for flowering time (DTF), growth habit (GH), and branching index (BI) under short (SD) and long days (LD). Numbers at the left of the bar indicate genetic distance (in cM). Markers included in the 3A interval (95% interval confidence) are highlighted in green and the most strongly associated marker (*FT*a1) is shown in red.

BI of the population was generally higher in SD than in LD, as might be expected in view of the longer vegetative growth phase. However, across the population, a strong positive correlation (*r* = 0.679, *p* < 0.001) was found in the BI between photoperiods, suggesting that at this stage (3 weeks old plants) a genetic component of this trait is unrelated to photoperiod. QTL analysis revealed two QTLs for growth habit; a major QTL on LG3 that explained 66% of the variation for this trait, and a minor QTL on LG4. For BI, a single QTL in a similar location was identified under both photoperiods (Table 1). Interestingly, the QTL for both GH and BI in chromosome 3 were closely co-located with the *DTF3A* flowering time QTL described above (Figure 4). In addition, the physiology of these three QTL is similar with respect to their strong effect under SD and their absence, or minor effect, under LD, as seen in the genotype means for the *FTa1* peak marker shown in Table 1.

## Discussion

One of the critical events in chickpea evolutionary history is thought to have been its conversion from a winter to a summer crop, likely achieved by Neolithic farmers in an attempt to reduce the incidence of Ascochyta blight, whose onset is favored by the cool, wet conditions that typify Mediterranean winters (Kumar and Abbo, 2001; Abbo et al., 2003a, b). For this shift in the chickpea farming system to succeed, a major modification of phenology toward earliness would have been required in order to match the considerably shorter growing season. This selective pressure is evident today in the typically early flowering phenotype of the domesticated *C. arietinum* relative to wild *Cicer* species (Berger J. et al., 2004).

Our analyses identify a central region of chromosome 3 (referred to as region 3A) that makes a major contribution to this difference in flowering time between domesticated chickpea and its wild progenitor, *C. reticulatum*, in two populations utilizing different *C. arietinum* parents and grown in different conditions. This result is consistent with several previous reports. Das et al. (2015) found a recurrent major QTL on chromosome 3 in an interspecific cross using ICC4958 as the domesticated parent. Aryamanesh et al. (2010) found a major QTL on chromosome 3 defined by the same interval as that reported initially in RIP12 by Cobos et al. (2009) and narrowed in the present study. The fact that these studies use different and unrelated *C. arietinum* accessions suggests that the presence of early alleles at this locus may be a defining feature of domesticated chickpea.

Another interpretation is that the apparent importance of this locus could reflect the fact that the wild parents used in all of these studies are closely related and could conceivably carry a unique variant at this locus that is not representative of the wider *C. reticulatum* germplasm. However, this is discounted by the recent finding of von Wettberg et al. (2018), who examined crosses between a common domesticated parent and 29 newly collected wild accessions representing a much wider diversity, and found that all progenies shared a common major QTL in a 3.55 Mb interval of chromosome 3 encompassing the LG3A region. Interestingly, this region also appears to have a significant effect within domesticated chickpea, as revealed by our analysis of two intraspecific populations, and several other studies (e.g., Hossain et al., 2010; Rehman et al., 2011). However, its effect at this level seems to be more dependent on environment and the influence of other loci, suggesting that additional variation in this region may have also had a role in post-domestication diversification of flowering behavior. Further clarification of this scenario will require a wider analysis in both interspecific and intraspecific contexts, whether in biparental populations or through association approaches.

In addition to late phenology, wild chickpea is also distinguished from domesticated forms by the greater profusion of branches and prostrate growth habit (Ali et al., 2015), and we found that the same chromosomal region 3A also had a significant influence on both traits, particularly under SD conditions, as reflected by the presence in the region of a major QTL for each of these traits (QTL *GH3* and QTL *BI3*). To date, two major loci, *Hg* and *Hg2*, have been reported to determine growth habit differences between *C. arietinum* and *C. reticulatum* (Muehlbauer and Singh, 1987; Kazan et al., 1993; Ali et al., 2015). Interestingly, *Hg* has been mapped to the central region of chromosome 3 by Winter et al. (2000), using a population derived from the same parents as CRIL2, and studies by Cobos et al. (2009), Aryamanesh et al. (2010), Ali et al. (2015) have all reported a locus influencing growth habit in this region. Since the *GH3* QTL we describe here for CRIL2 is located within the intervals reported in these studies, it seems likely that all of these studies are detecting the same locus (*Hg*). Association of flowering with different features of shoot architecture has been previously described in a number of other legume species, including chickpea (Lichtenzveig et al., 2006; Julier et al., 2007; Lagunes Espinoza et al., 2012; González et al., 2016; Yang et al., 2017). In the case of QTL in the chickpea LG3A region, such an association could either represent the action of independent but tightly linked genes, or the pleiotropic effects of a single gene.

The discrete and approximately 1:1 segregation of flowering time in CRIL2 under controlled SD conditions enabled us to map *DTF3A* as a Mendelian trait to a narrower interval, thereby reducing the number of potential candidates. The only remaining clear candidates were a cluster of three *FT* genes orthologous to the *FTa1*/*a2*/*c* cluster identified in Medicago and pea by Hecht et al. (2005, 2011). *FT* genes have a widely conserved role as flowering promoters (Wickland and Hanzawa, 2015), and several recent studies show that this is also the case for legume *FTa* and *FTc* genes (Kong et al., 2010; Hecht et al., 2011; Laurie et al., 2011; Sun et al., 2011). We identified elevated expression of genes in the *FT* cluster in the early parents of all three crosses examined (Figure 3), implicating the general de-repression of these genes as the likely molecular basis for the *DTF3A* effect. A comparable situation has been recently described in another legume, narrow-leafed lupin (*Lupinus angustifolius*), where a strong ancestral vernalization requirement has restricted production in warmer regions. This limitation has been overcome by the incorporation of dominant alleles at the major locus *Ku*, which confer de-repressed expression of a tightly linked *FTc* gene and permit flowering in the absence of vernalization (Nelson et al., 2017; Taylor et al., 2018). However, compared to lupin, where only a single *FT* gene is present in this genomic location, the presence of three genes in chickpea is clearly a more complex situation, and raises the question of which of them might be responsible for the QTL effects on photoperiod response, or the QTLs for vernalization response that has been localized to the same genomic region on LG3 (Samineni et al., 2015; Pinhasi van-Oss et al., 2016).

The *FTa1* gene plays a key role in regulation of flowering in both pea and Medicago, as loss-of function mutants show significant impairment of flowering in both species, and overexpression in Medicago confers early flowering and reduced sensitivity to photoperiod and vernalization (Hecht et al., 2011; Laurie et al., 2011). *FTa1* would therefore seem to be the strongest candidate for the causal gene underlying *DTF3A*. Although the role of *FTc* has not been systematically explored in either species, both *MtFTc* and *PsFTc* are strong activators of flowering when overexpressed in Arabidopsis, and their induction in apical tissues correlates closely with flowering (Hecht et al., 2011), suggesting that the higher levels of *CaFTc* expression could also potentially contribute to the earlier flowering of domesticated lines. Intriguingly, the most dramatic expression difference in the two interspecific comparisons was seen for *FTa2*, which was expressed at a low level in *C. reticulatum* parents and over 20 times higher in the domesticated parents. However, despite this striking association with early flowering, *FTa2* was not expressed at all in the early parent of the intraspecific cross, indicating that the early flowering of domesticated relative to wild chickpea cannot result primarily from the high level of *FTa2* expression. Also, in contrast to *FTa1* and *FTc*, *FTa2* from pea or Medicago is much less effective for induction of flowering when expressed in transgenic Arabidopsis, and its endogenous expression patterns are not consistently associated with flowering (Hecht et al., 2011; Laurie et al., 2011). Taken together, these observations suggest that *FTa2* is less likely to be the basis for the interspecific effects of *DTF3A*, but it remains plausible that these effects might reflect general de-repression across the cluster and a functional contribution from all three genes.

The strong photoperiod-dependence of the *DTF3A* effect can also be interpreted in terms of the known role of *FT* genes in mediating of environmental effects on flowering. In both pea and Medicago, photoperiod and vernalization responses appear to be integrated through *FT* genes, but whereas *FTa* genes are regulated by both photoperiod and vernalization, *FTb* genes are strictly regulated by photoperiod (Hecht et al., 2011; Laurie et al., 2011). In chickpea, a similar LD-specific expression of the single *FTb* gene is seen in both wild and domesticated parents (Figure 3) and may be sufficient for maximal promotion of flowering, which could provide an explanation for the minimal effect of *DTF3A* under these conditions. In contrast, under non-inductive SD conditions, the absence of *FTb* expression or other inputs would presumably expose any effects of elevated expression of the *FTa/c* cluster.

Whether one or more of the *FT* genes are indeed responsible for the effects of *DTF3A*, it is also of interest to consider what might be the molecular basis of their observed de-repression. The apparently specific effects of the QTL on expression of the underlying *FT* genes suggests a scenario in which the domesticated parents might have undergone modification of either a *cis*-acting or a closely linked *trans*-acting mechanism normally required for repression of the cluster. The absence of other plausible candidates in the defined region favors a *cis*-acting mechanism, and precedent for this is provided by recent studies in two other legumes. In Medicago, insertions in the third intron and 3′ flanking region of *FTa1* confer gain-of-function phenotypes, with elevated *FTa1* expression and dominant early flowering (Jaudal et al., 2013), whereas in narrow-leafed lupin, the derepression of *FTc* expression that underlies the effects of *Ku* alleles is associated with deletions in the *FTc* promoter (Nelson et al., 2017; Taylor et al., 2018). The recently reported role for the polycomb-group protein VRN2 (*VERNALIZATION 2*) in *FTa1* repression in Medicago (Jaudal et al., 2016) points to the likely existence of both epigenetic and transcriptional components to this regulation.

Direct involvement of *FT* genes would also provide an explanation for the association of growth habit and flowering effects with the chromosome 3A region. It is becoming increasingly apparent that *FT* genes, in addition to being major flowering regulators, also affect plant architecture and growth habit across a wide range of plant species including Arabidopsis, tomato, rose and rice (Lifschitz et al., 2006; Tamaki et al., 2007; Hiraoka et al., 2013; Huang et al., 2013; Randoux et al., 2014; Tsuji et al., 2015; Weng et al., 2016). However, the most direct and relevant comparison with chickpea is again provided by Medicago, where *MtFTa1* overexpression converts the prostrate habit of plants grown under SD to a more erect habit typical of LD (Laurie et al., 2011). This effect is clearly similar to that of the corresponding region on chromosome 3A in domesticated chickpea. In contrast, Medicago *fta1* mutants show a highly branched, prostrate phenotype under LD similar to that of wild-type under SD, further emphasizing the multiple roles of *FTa1*. This observation strengthens the case that the major flowering time and growth habit loci in this region of chromosome 3 represent pleiotropic effects of misexpression of genes in the *FT* cluster, and possibly of *FTa1* in particular.

An emerging theme in long day legumes appears to be an important adaptive role for dominant genetic variants in the region of the *FTa/c* cluster that relax the environmental constraints on flowering and permit early flowering (Weller and Ortega, 2015). Whether a common molecular mechanism unites these adaptations and explains their repeated evolution remains to be determined. Among the ancient legume crops, chickpea in particular may represent a unique example in which modification of such a mechanism has been fundamentally important to crop success. Future, more detailed analyses should shed light on its molecular basis and physiological consequences, and its significance for chickpea domestication and adaptation.

## Author Contributions

JW, RO, VH, and TM conceived the study. JW and RO designed the study. RO, RM, NC-G, RP, JR, and TM carried out the experiments and/or generated the data. RO and JW wrote the manuscript with inputs from the other authors. All authors analyzed the data.

## Conflict of Interest Statement

The authors declare that the research was conducted in the absence of any commercial or financial relationships that could be construed as a potential conflict of interest.

## Footnotes

1 https://www.coolseasonfoodlegume.org
