# Supplementary material for: Altered Expression of an FT Cluster Underlies a Major Locus Controlling Domestication-Related Changes to Chickpea Phenology and Growth Habit
Source: Front Plant Sci. 2019 Jul 3;10:824. doi: 10.3389/fpls.2019.00824 (PMC6616154; doi:10.3389/fpls.2019.00824)
Supplement: Supplementary file 1 [file Data_Sheet_1.PDF]

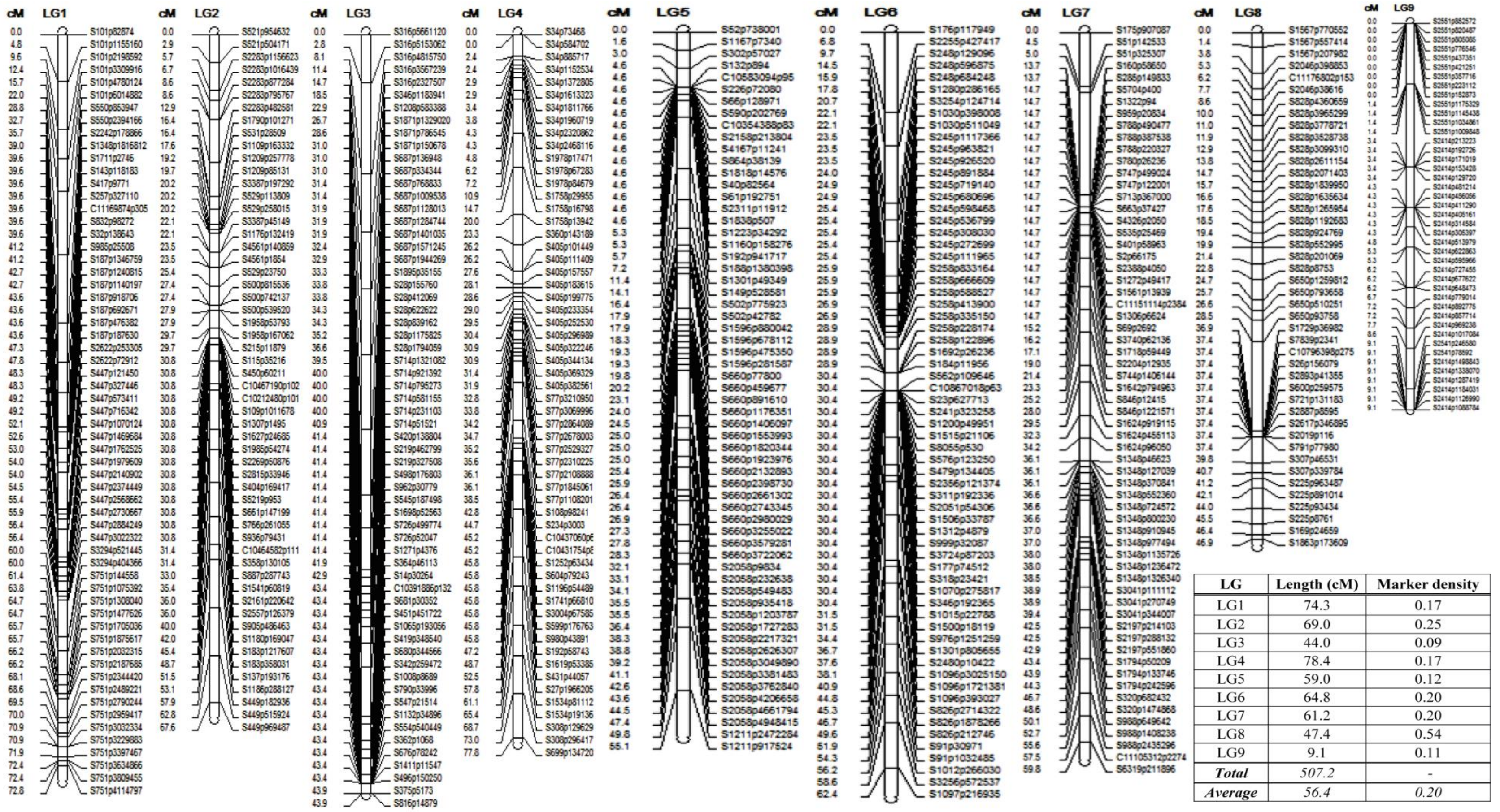

LG	Length (cM)	Marker density
LG1	74.3	0.17
LG2	69.0	0.25
LG3	44.0	0.09
LG4	78.4	0.17
LG5	59.0	0.12
LG6	64.8	0.20
LG7	61.2	0.20
LG8	47.4	0.54
LG9	9.1	0.11
Total	*507.2*	-
Average	*56.4*	*0.20*

Supplementary Figure 1. High density linkage map of the chickpea interspecific population CRIL2, ICC4958 (*Cicer arietinum*) x PI489777 (*Cicer reticulatum*), which was used to select a subset of markers to contruct genetic maps with the HRM markers designed in this study (Figure S4). The high density map is based on 2956 RAD-GBS SNP markers genotyped in 107 RIL lines (von Wettberg et al. 2018). It comprised nine linkage groups, with a total map length of 507.2cM. The map length of each linkage group varied from 9.1cM (LG9) to 78.4cM (LG4) with an average of 56.36cM (Inset table). A total of 2,956 markers were mapped on all of the 9 linkage groups. The number of markers mapped on an individual linkage group varied from 86 (LG9) to 495 (LG3) with an average of 328.44 markers/LG.

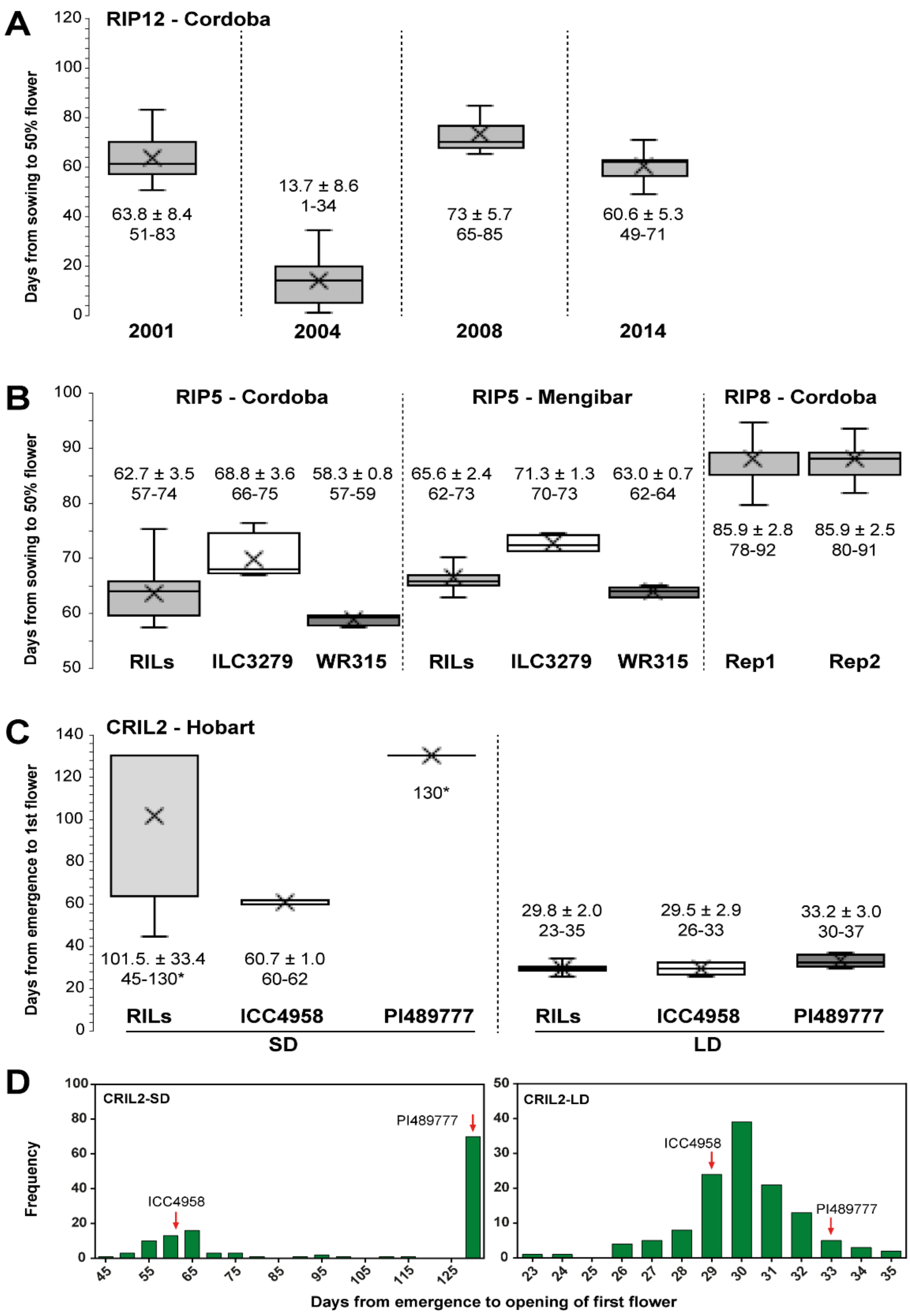

Supplementary Figure 2. Boxplot illustrating the variation in flowering time obtained in different environments for RIP12 **(A)**, RIP5/RIP8 **(B)** and CRIL2 **(C)**. Note that for RIP12, the flowering values during the 2004 season are relative: the date of the first visible flower in any RIL of the population was considered as day 1 and used as reference for the rest of the population. Box edges denote the lower and upper quantiles with mean (cross) and median (line) values in the middle of the box. Numbers under the boxes correspond to mean ± standard deviation and range (minimum-maximum value). **(D)** Frequency distribution of flowering time variation measured in CRIL2 parents and RILs grown under long (LD) and short days (SD). Red arrows indicate the phenotypic values of the parental lines.

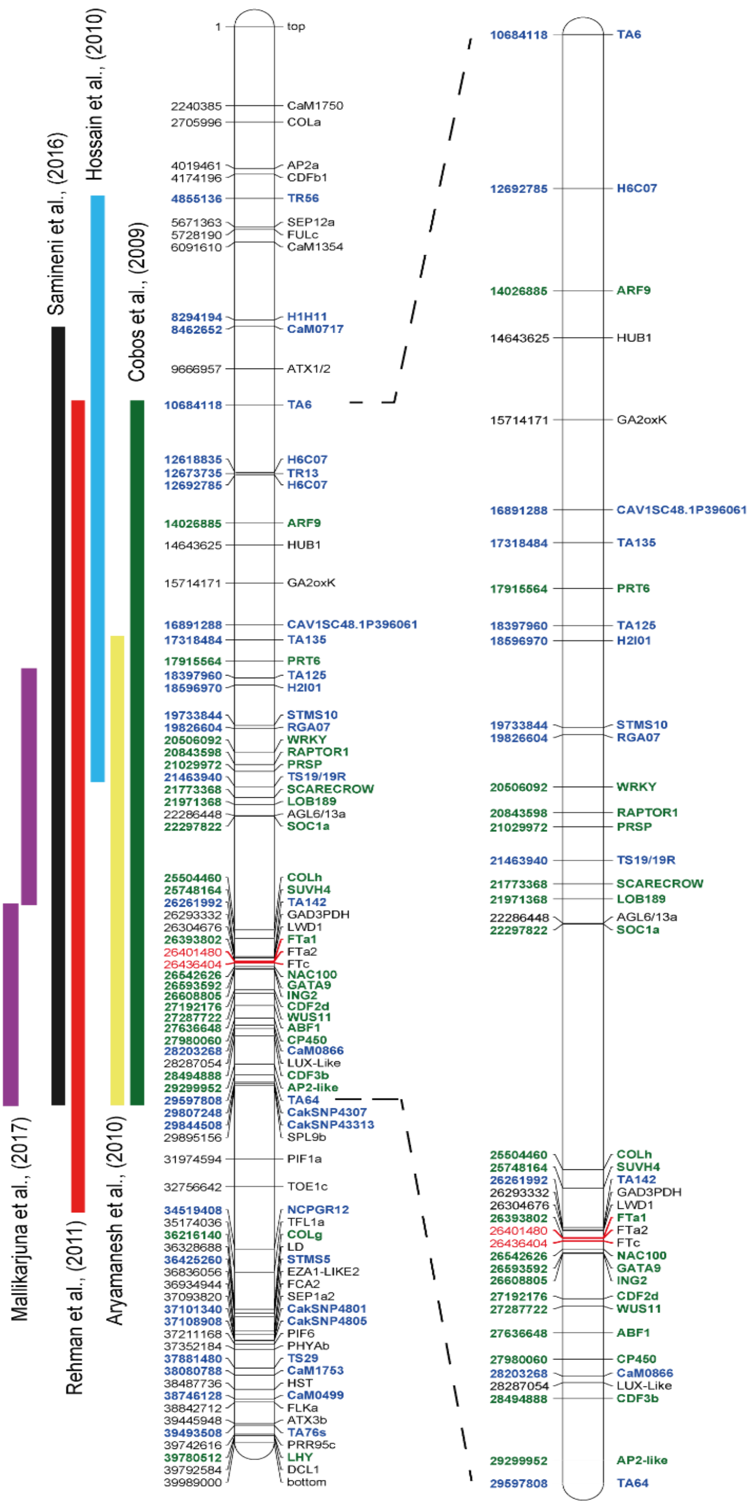

Supplementary Figure 3. Representation of the chickpea chromosome 3 and the region between markers TA6 and TA64, summarizing the different flowering QTL (vertical coloured bars) described to date in the region (Aryamanesh et al. 2010; Cobos et al. 2009; Mallikarjuna et al. 2017; Rehman et al. 2011; Samineni et al. 2016). Numbers on the left of the linkage group indicate physical position in bp, according to the genome assembly of cultivar CDC Frontier (ASM33114v1; Varshney et al., 2013). Interesting markers from previous reports are shown in blue and markers designed for this study are shown in green. Other interesting flowering-related genes are shown in black while the cluster of *FT* genes is highlighted in red.

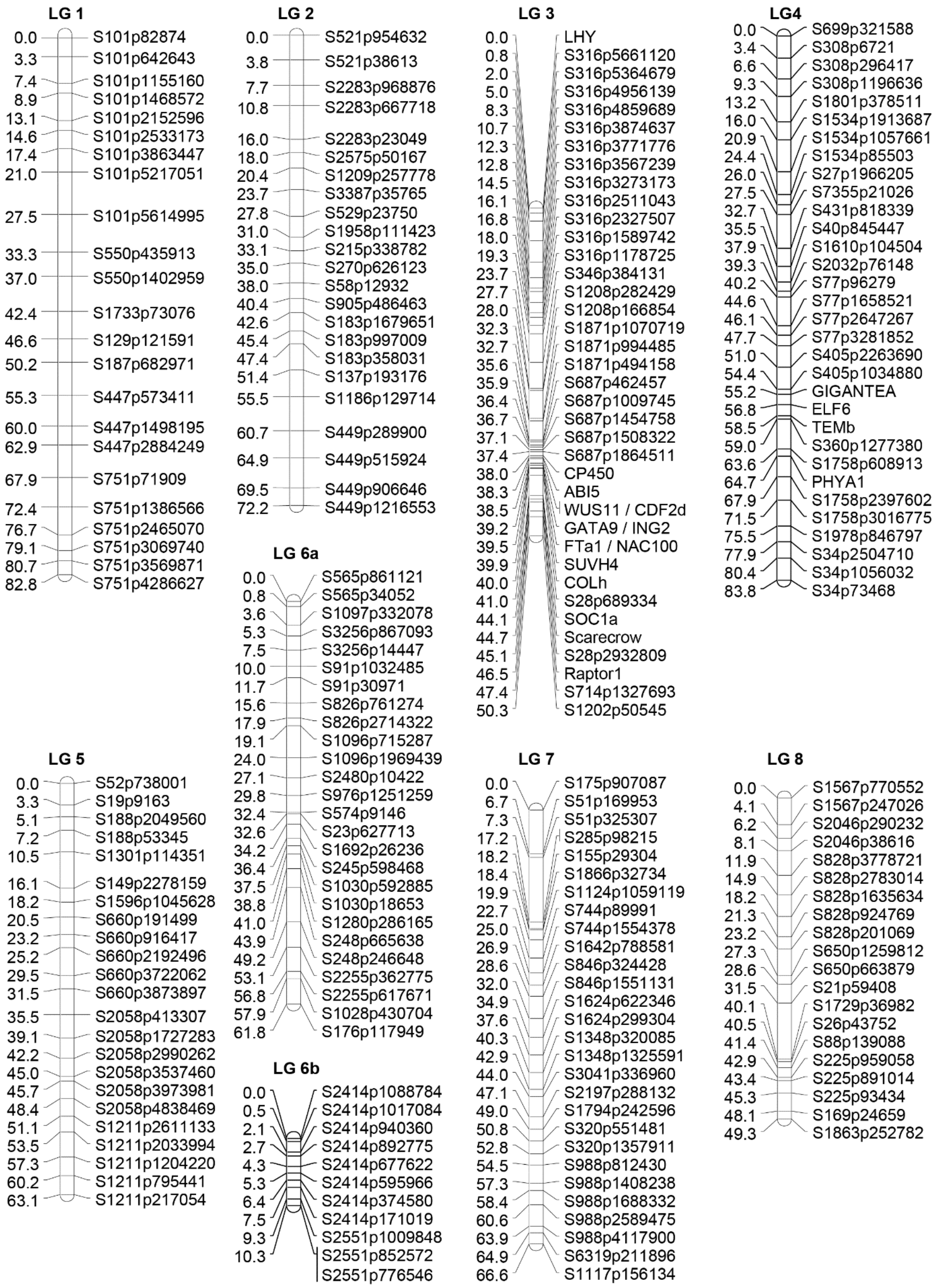

Supplementary Figure 4. Genetic linkage map constructed for CRIL2 (ICC4958 x PI489777). Numbers on the left of each linkage group represent the absolute distances in cM. The map comprises a total of 226 markers, grouped in 9 linkage groups covering a total distance of 540 cM, with an average inter-maker distance of 2.4 cM.

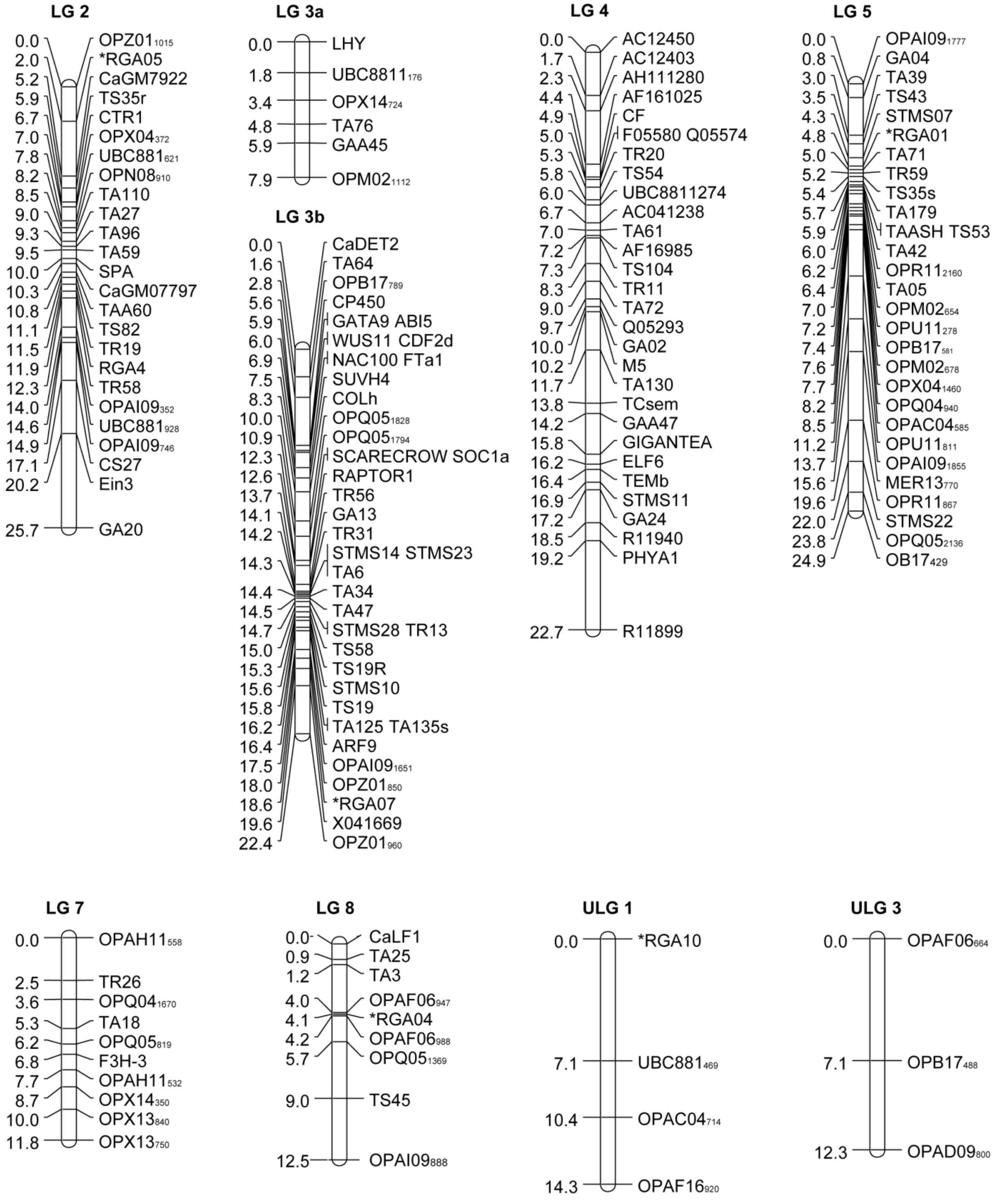

Supplementary Figure 5. Genetic linkage map constructed for RIP12 (ICCL81001 x Cr5-9). Numbers on the left of each linkage group represent the absolute distances in cM. The map comprises a total of 155 markers, grouped in 9 linkage groups covering a total distance of 154.5 cM, with an average inter-maker distance of 1.05 cM.

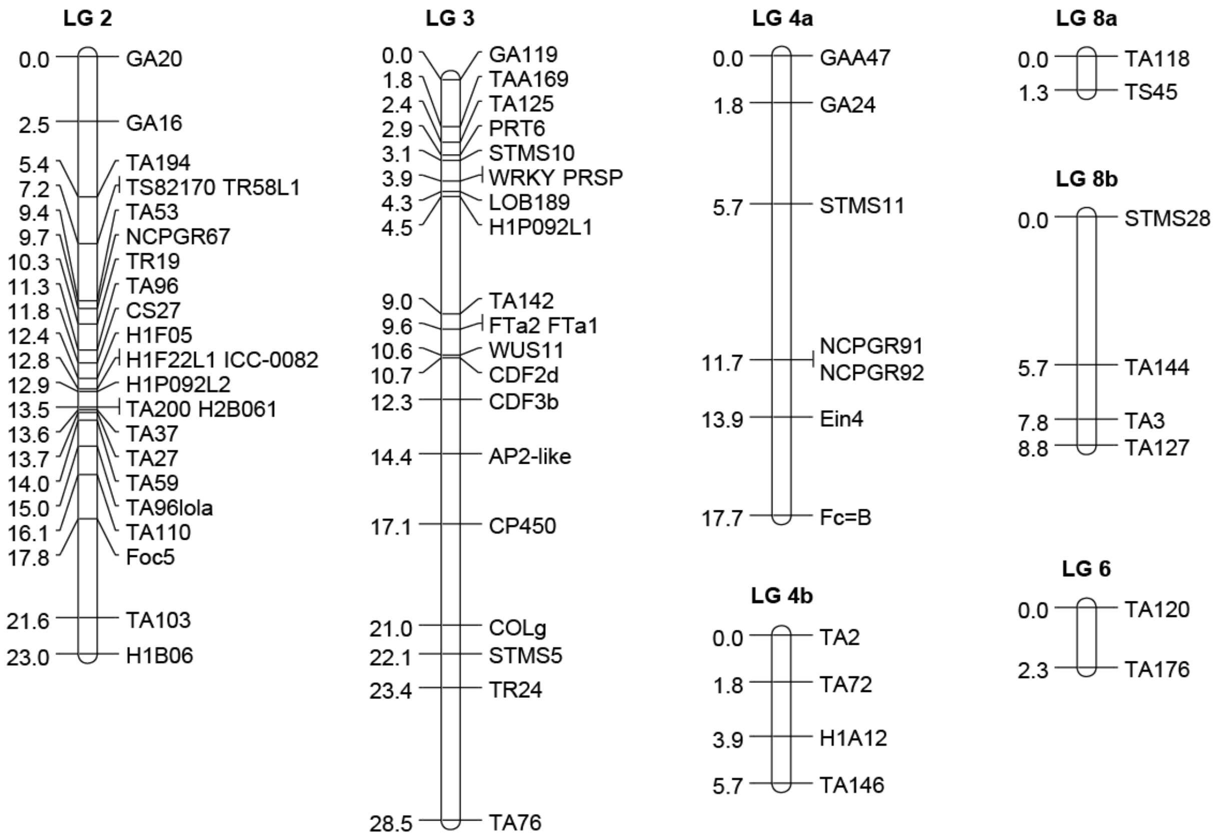

Supplementary Figure 6. Genetic linkage map constructed for RIP5 (WR315 x ILC3279). Numbers on the left of each linkage group represent the absolute distances in cM. The map comprises a total of 64 markers, grouped in 7 linkage groups covering a total distance of 87.3 cM, with an average inter-maker distance of 1.53 cM.

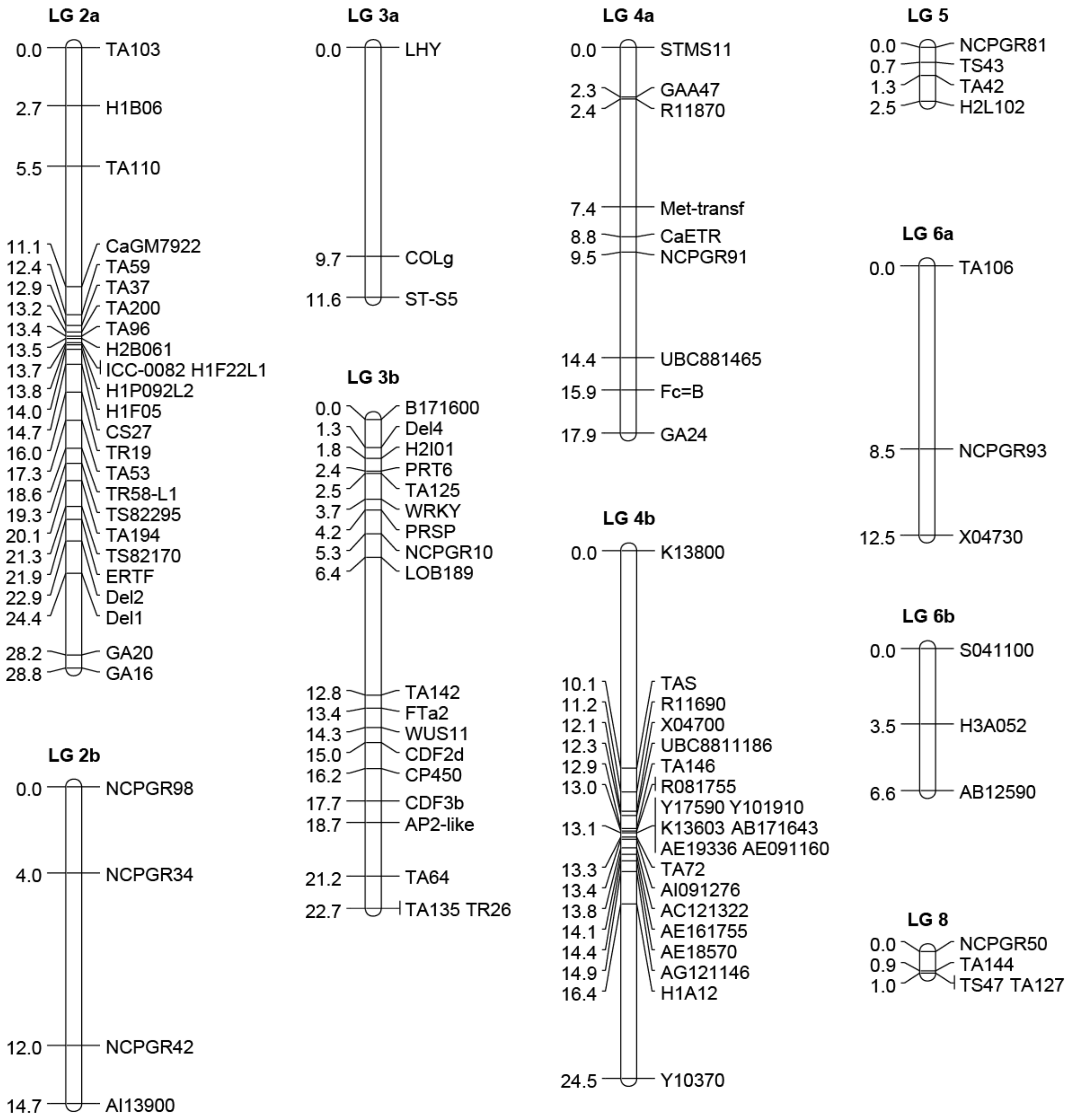

Supplementary Figure 7. Genetic linkage map constructed for RIP8 (ILC3279 x WR315). Numbers on the left of each linkage group represent the absolute distances in cM. The map comprises a total of 95 markers, grouped in 10 linkage groups covering a total distance of 142.7 cM,with an average inter-maker distance of 1.76 cM.

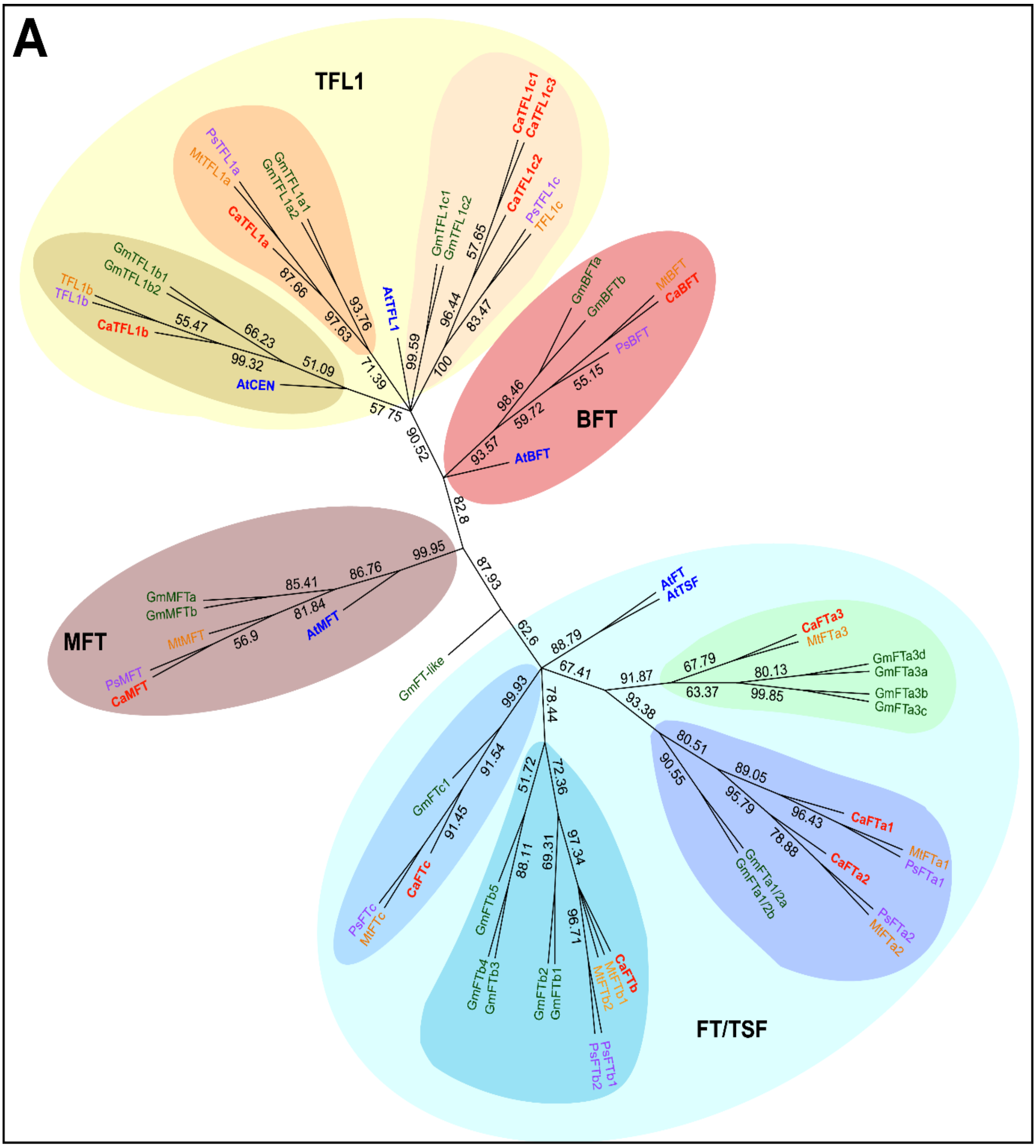

Supplementary Figure 8. (**A**) Maximum parsimony tree derived from the alignment of phosphatidylethanolamine-binding proteins **(**PEBP) in *Arabidopsis thaliana* (At, blue), *Cicer arietinum* (Ca, red), *Glycine max* (Gm, green), *Pisum sativum* (Ps, purple) and *Medicago truncatula* (Mt, orange). Accession number of the PEBP genes can be found in Supplementary Table 4. Sequences were aligned with MAFFT (full alignment can be found in Supplementary Figure 9) and the tree was build using PAUP* in Geneious 8 software. Numbers beside branches represent bootstrap support from 1000 replications. (**B**) In next page; Position of the PEBP genes (in red) in the chickpea reference genome. Bars represent chromosomes, with numbers indicated on top. *BFT* and *TFL1c1* are not included as they were located in an unplaced scaffold (NW_004516558.1 and NW_004516523.1, respectively).

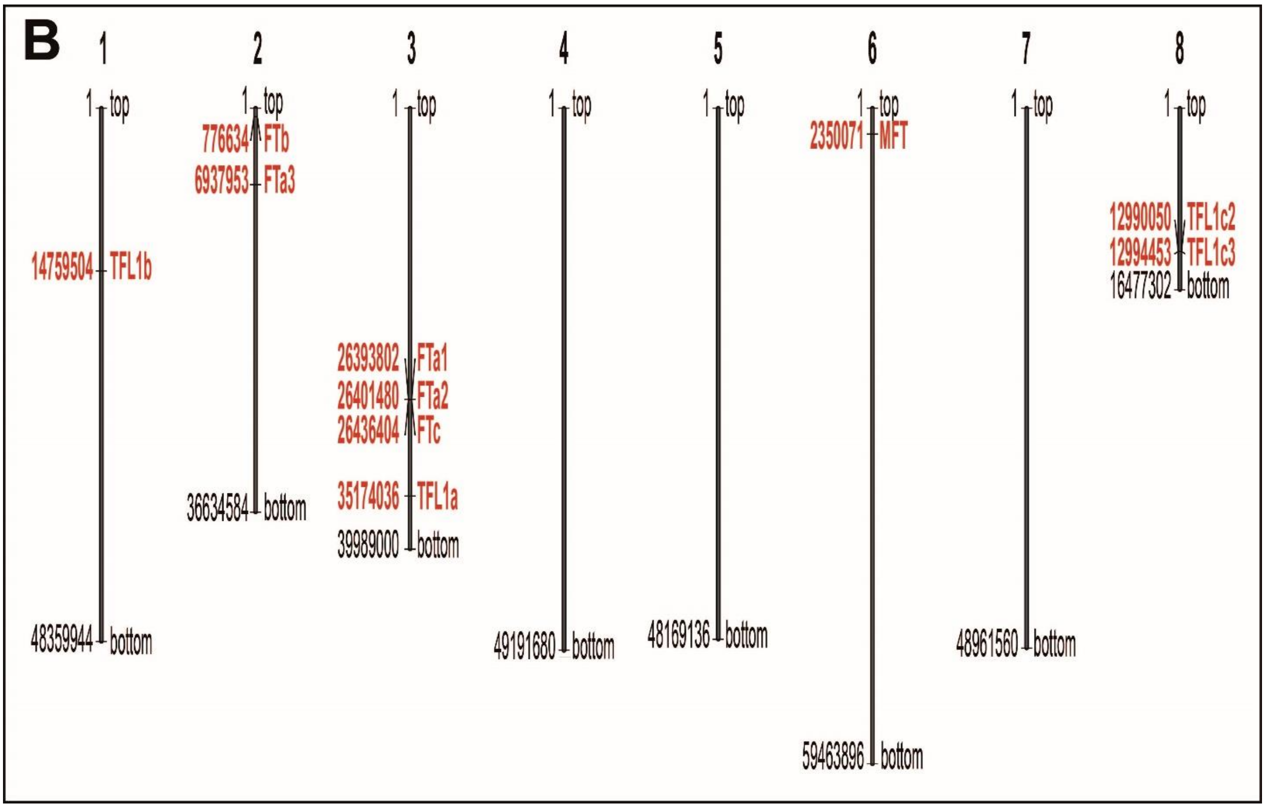

Supplementary Figure 8 B.

Supplementary Figure 9. Multiple sequence alignment of the PEBP sequences from five species described in Supplementary Table 1. Proteins were aligned in Geneious 8 using MAFFT. Residues conserved in more than 80% of the sites are presented with black background/while letters. Those with a conservation between 60-80% show grey background/white letters and those between 40-60% with grey background/black letters.

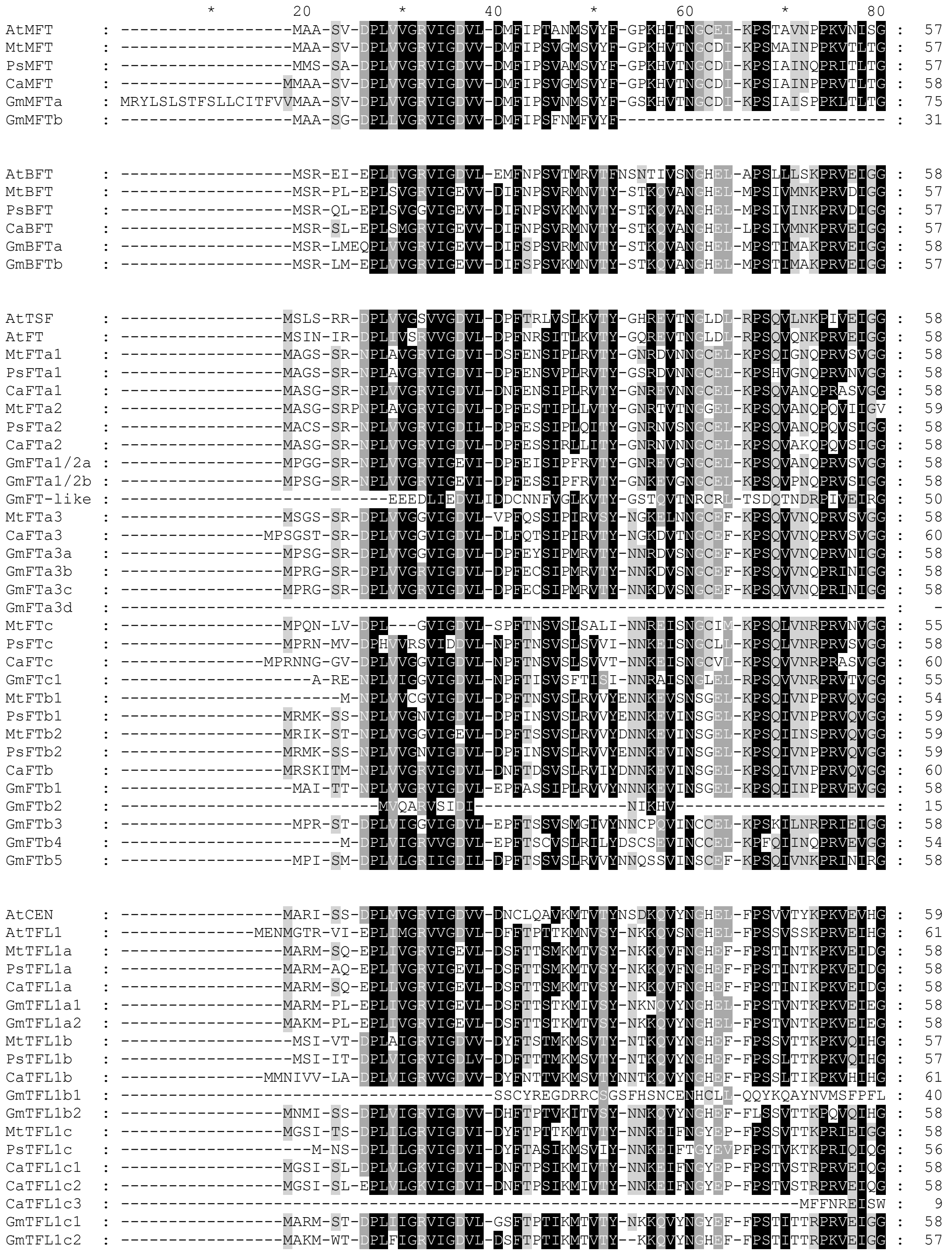

Supplementary Figure 9. Continued

```
                     *      100       *      120       *      140       *      160
AtMFT      : -HSDELYTLVMTDPDAPSPSEPNMREWVHWIVVDIPGG------------TNPSRGKEILPYMEPRPPVGIHRYILVLFR : 124
MtMFT      : -NMDNLYTLVMTDPDAPSPSEPSMRELIHWIVVDIPGG------------TNPKRGKEILPYIGPKPPVGIHRYILVLFE : 124
PsMFT      : -NRSSLYTLVMTDPDAPSPSEPSLREFIHWIVVDIPGG------------TNPKRGHEILPYIGPKPPVGIHRFILVLFE : 124
CaMFT      : -NIDNLYTLVMTDPDAPSPSEPSMRELIHWIVVDIPGG------------TNPKRGKEILPYIGPKPPVGIHRFILVLFK : 125
GmMFTa     : -NMDNLYTLVMTDPDAPSPSEPSMREWIHWILVDIPGG------------TNPFRGKEIVSYVGPRPPIGIHRYIFVLFQ : 142
GmMFTb     : -----------------GPSEPSMREWIHWIVVDILEEQTHFVFHDVACMSTGGSRKEIVPYLGPRPPIGIHRYIFLLFQ :  94

AtBFT      : QDLRSFFTLIMMDPDAPSPSNPYMREYLHWMVTDIPGT------------TDASFGREIVRYETPKPVAGIHRYVFALFK : 126
MtBFT      : EDMRSAYT-IMTDPDAPSPSDPHLREHLHWMVTDIPGT------------TDVSFGNEIVEYENPKPVIGIHRYVFILFK : 124
PsBFT      : DDMRSAYTLVMTDPDAPSPSDPYLREHLHWMVADIPGT------------TDVSFGKEIVEYENPKPVIGIHRYVFILFK : 125
CaBFT      : DDLRTAYTLIMTDPDAPSPSDPYLREHLHWMVTDIPGT------------TDVSFGKEIVEYENPKPVIGIHRYVFILFK : 125
GmBFTa     : DDMRTAYTLIMTDPDAPSPSDPHLREHLHWTVTDIPGT------------TDVSFGKEIVGYESPKPVIGIHRYVFILFK : 126
GmBFTb     : DDMRTAYTLIMTDPDAPSPSDPCLREHLHWMVTDIPGT------------TDVSFGKEIVGYESPKPVIGIHRYVFILFK : 125

AtTSF      : DDFRNFYTLVMVDPDVPSPSNPHQREYLHWLVTDIPAT------------TGNAFGNEVVCYESPRPPSGIHRIVLVLFR : 126
AtFT       : EDLRNFYTLVMVDPDVPSPSNPHLREYLHWLVTDIPAT------------TGTTFGNEIVCYENPSPTAGIHRVVFILFR : 126
MtFTa1     : NDLRNLYTLVMVDPDSPSPSNPTFKEYLHWLVTDIPGT------------TEVTFGNEVVNYERPRPTSGIHRFVFVLFR : 126
PsFTa1     : NDLRNIYTLVLVDPDSPSPSNPTFREYLHWLVTDIPAT------------TEVSFGNEIVSYERPRPTSGIHRFVFILFR : 126
CaFTa1     : NDMRNFYTLVLVDPDSPSPSNPTFREYLHWLVTDIPAT------------TGVSFGNEVVGYERPRPTSGIHRFVFVLFR : 126
MtFTa2     : NDPTALYTLVLVDPDAPSPSYPSFREYLHWMVTDIPAT------------NAASFGNEVVSYEKPRPNLGIHRFVFVLLH : 127
PsFTa2     : NDPVIYYTLVLVDPDAPSPSYPSFREYLHWMVTDIPAT------------TGASFGNEVVSYEKPRPNLGIHRFVFVLLR : 126
CaFTa2     : NDLRIFYTLVLVDPDAPSPSNPSFREYLHWLVTDIPST------------AGASFGNEVVCYEKPRPNLGIHRYVFVLFR : 126
GmFTa1/2a  : DDLRNFYTMVLVDPDAPSPSNPNFREYLHWLVTDIPET------------TGPNFGNEVVSYESPRPTMGIHRLVFVLFR : 126
GmFTa1/2b  : DDLRKFYTMVMVDPDAPSPSNPNFREYLHWLVTDIPET------------TGPNFGNEIVSYESPRPTMGIHRFVFVLFR : 126
GmFT-like  : -DANSFYTLVMVDPDSPSRDKPTEREHLLCQYSSRGSNLRY-------VLTQFFFGEEVVPYEGPFPHRWIHRIVFVLFR : 122
MtFTa3     : DDLRNFYTLIMVDPDAPSPSNPNLREYLHWLVTDIPAT------------TGPTFGHEVVPYESPRPSMGIHRIVFVIFR : 126
CaFTa3     : DDLRNFYTLIMVDPDAPSPSNPNLREYLHWLVTDIPAT------------TGPTFGNEVVTYENPRPFMGIHRIIFVVFQ : 128
GmFTa3a    : DDLRNFYTLIAVDPDAPSPSDPNLREYLHWLVTDIPAT------------TGASFGHEVVTYESPRPMMGIHRLVFVLFR : 126
GmFTa3b    : DDFRNFYTLIAVDPDAPSPSDPNFREYLHWLVTDIPAT------------TGPTFGHEVVTYENPRPMMGIHRIVFVLFR : 126
GmFTa3c    : DDFRNFYTLIAVDPDAPSPSDPNFREYLHWLVTDIPAT------------TGPTFGHEVVTYENPRPMMGIHRIVFVLFR : 126
GmFTa3d    : -------------------------------------------------------------------MMGIHRLVFVLFR :  13
MtFTc      : DDLRTFYTMVMVDADAPSPSNPFLKEYLHWMVTDIPAT------------TSASFGKEVVFYESPKPSAGIHRFVIALFK : 123
PsFTc      : EDLRTFYTLAMVDADAPSPSNAFLREYLHWMVTDIPAT------------TSASFGKEAVFYESPKPSAGIHRFVIVLFK : 126
CaFTc      : EDLRTFYTLVMVDADAPSPSNPVLREYLHLMVTDIPAT------------TSANFGKEVVFYESPNPSAGIHRLVIVLFK : 128
GmFTc1     : EDLRTFYTLVMVDADAPSPSNPVLREYLHWMVTDIPAT------------TNASFGREVVFYESPNPSAGIHRLVFILFQ : 123
MtFTb1     : NDLRTLYTLVMVDPDGPSPSNPNMREYLHWMVTNIPAT------------TGTTFGQEIVSYENPRPTSGIHRVIFVLFR : 122
PsFTb1     : NDFRTLYTLVMVNPDAPSPCNPHMREYLNWMVTNIPAT------------TGTTFGQEIVSYESPRPTSGIHRIIFVLFQ : 127
MtFTb2     : NDLRTLYTLVMVNPDAPSPSDPNMREYLYWMVTNIPAT------------TGTTFGQEIVSYESPRPASGIHRVIFVLFR : 127
PsFTb2     : NDFRTLYTLVMVNPDAPSPSDPNMREYLYWMVTNIPAT------------TGTAFGQEIVSYESPRPASGIHRMIFVLFQ : 127
CaFTb      : NDFRTLYTLVMVDPDAPSPSDPNMREYLYWMVTNIPAT------------TGTTFGQEIVSYENPRPTSGIHRVVFVLFR : 128
GmFTb1     : DDLRTLYTLVMVDPDAPSPSDPNMREYLHWLVTNIPAT------------TSASFGQEVVSYESPRPTSGIHRFIFVLFR : 126
GmFTb2     : -------------------------------------R------------TVILIGQEVVSYESLQPTSGIHQ------- :  39
GmFTb3     : DDLRTFYTLVMVDPDAPSPGNPTQREYLHWLITNIPAT------------TGANFGEEIVSYESPRPIVGIHRIVFVLFR : 126
GmFTb4     : DDFRTFYTLVMVDPDAPSPGNPNQREYLHWLVTNIPGT------------TGANFGEEVVSYESPRPMMGIHRIIFILFR : 122
GmFTb5     : NDLGIFYTLIMVNPDAPSPSDPHMKEYLHWLVTNIPAS------------TGATTGEEIVEYESPRPTSGIHRIAFVLFR : 126

AtCEN      : GDMRSFFTLVMTDPDVPGPSDPYLREHLHWIVTDIPGT------------TDVSFGKEIIGYEMPRPNIGIHRFVYLLFK : 127
AtTFL1     : GDLRSFFTLVMIDPDVPGPSDPFLKEHLHWIVTNIPGT------------TDATFGKEVVSYELPRPSIGIHRFVFVLFR : 129
MtTFL1a    : GDMRSFYTLVMTDPDVPGPSDPYLREHLHWIVTDIPGT------------TDATFGKEVVSYEIPKPNIGIHRFVFVLFK : 126
PsTFL1a    : ADMRSFYTLVMTDPDVPGPSDPYLREHLHWIVTDIPGT------------TDATFGKEIVSYEIPKPNIGIHRFVFVLFK : 126
CaTFL1a    : GDMRSFFTLVMTDPDVPGPSDPYLREHLHWIVTDIPGT------------TDATFGKELVSYEIPKPNIGIHRFVFVLFK : 126
GmTFL1a1   : GDMRSFFTLIMTDPDVPGPSDPYLREHLHWIVTDIPGT------------TDATFGKELVSYEIPKPNIGIHRFVFVLFK : 126
GmTFL1a2   : GDMRSFFTLIMTDPDVPGPSDPYLREHLHWIVTDIPGT------------TDATFGKELVSYEVPKPNIGIHRFVFVLFK : 126
MtTFL1b    : GDMRSFFTLVMTDPDVPGPSDPYLKEHLHWIVTDIPGT------------TDATFGKEVMKYEMPRPNIGIHRFVFLLYK : 125
PsTFL1b    : GDMRSFFTLIMTDPDVPGPSDPYLKEHLHWIVTDIPGT------------TDATFGKEVMKYEMPRPQIGIHRFVFLLYK : 125
CaTFL1b    : GDMRSFFTLIMTDPDVPGPSDPYLKEHLHWIVTDIPGT------------TDATFGKEVIKYEMPRPNIGIHRFVFILYK : 129
GmTFL1b1   : SDMRSFFTLVMTDPDVPGPSDPYLREHLHWLVTDIPGT------------TDATFGNEVVEYEIPRPNIGIHRFVFLVFK : 108
GmTFL1b2   : GDMRSFFTLVMTDPDVPGPSDPYLREHLHWMVTDIPGT------------TDATFGNEVVEYEILRPNIGIHRFVFLVFK : 126
MtTFL1c    : VDMRSLFTLIMIDPDVPGPSDPYMKEHLHWMVTDIPGT------------TDSTFGKELTSYEKPKPNIGIHRYVFVLFK : 126
PsTFL1c    : GDMRSLFTLIMIDPDVPGPSDPYMKEHLHWMVTDIPGT------------TDSTFGKELTSYEKPKPNIGIHRYVFVLFK : 124
CaTFL1c1   : GDMRSLFTLIMIDPDVPGPSDPYMREHLHWMVTDIPGT------------TDSTFGKELTSYEIPKPNIGIHRYVFVLFK : 126
CaTFL1c2   : GDMRSLFTLIMIDPDVPGPSDPYMREHLHWMVTDIPGT------------TDSTFGKELTSYEIPKPNIGIHRYVFVLFK : 126
CaTFL1c3   : NFLFIFFLQIMIDPDVPGPSDPYMREHLHWMVTDIPGT------------TDSTFGKELTSYEIPKPNIGIHRYVFVLFK :  77
GmTFL1c1   : GDMRSFYTLIMTDPDVPGPSDPYLREHLHWMVTDIPGT------------TNASFGKVLVSYEMPNPNIGIHRYVFVLLK : 126
GmTFL1c2   : GDMRSFYTLIMTDPDVPGPSDPYLREHLHWMVTDIPGT------------TNASFGNVLVSYEMPKPNIGIHRYVFVLFK : 125
```

Supplementary Figure 9. Continued

```
                       *        180         *        200         *
AtMFT       : QNS--PVGLMVQQPPSRANF-STRMFAGHFDLGLPVATVYFNAQKEPASRRR---- : 173
MtMFT       : QKG--PIGM-VEQPTSRVSF-NTRYFASQMNLGLPVATVYFNSQKEPQAKRR---- : 172
PsMFT       : QKG--PIGL-VEEPPSRVSF-NTRYFASQLDLGLPVATVYFNSQREPQSKRR---- : 172
CaMFT       : QKG--PMGL-VEQPTSRVSF-NTRYFASQLDLGLPVATVYFNSQKEPQAKRR---- : 173
GmMFTa      : QKG--PLGL-VEQPPTRASF-NTRYFARQLDLGLPVATVYFNSQKEPAVKRR---- : 190
GmMFTb      : QKV--PLGL-VEQPPTRASF-NTRYFVRQLDLGLPVATVYFNSQKEPAAKRR---- : 142

AtBFT       : QRG--RQAV-KAAPETRECF-NTNAFSSYFGLSQPVAAVYFNAQRETAPRRRPSY- : 177
MtBFT       : QRG--RQTV--RSPSSRDNF-NTRRFSQENNLGLPVAAVYFNAQRETAARRR---- : 171
PsBFT       : QRG--RQTV--RAPNSRDQF-STRIFSEENNLGLPVAAVYFNAQRETAARRR---- : 172
CaBFT       : QRG--RQTV--RSPTFRDNF-NTRSFSQQNSLGLPVAAVYFNAQRETAARRR---- : 172
GmBFTa      : QRG--RQTV--RPPSSRDHF-NTRRFSEENGLGLPVAVVYFNAQRETAARRR---- : 173
GmBFTb      : QRG--RQTV--RPPSSRDHF-NTRRFSEENGLGLPVAAVYFNAQRETAARRR---- : 172

AtTSF       : QLG--RQTV--YAPGWRQQF-NTREFAEIYNLGLPVAASYFNCQRENGCGGRRT-- : 175
AtFT        : QLG--RQTV--YAPGWRQNF-NTREFAEIYNLGLPVAAVFYNCQRESGCGGRRL-- : 175
MtFTa1      : QQC--RQRV--YAPGWRQNF-NTREFAELYNLGSPVAAVFFNCQRESGSGGRTFR- : 176
PsFTa1      : QQC--RQRV--YAPGWRQNF-NTREFAELYNLGSPVAAVFFNCQRESGSGGRTFR- : 176
CaFTa1      : QQC--RQRV--YAPGWRQNF-NTREFAELYNLGLPVAAVFFNCQRESGSGGRTFR- : 176
MtFTa2      : QQC--RQRV--YAPGWRQNF-NTREFIEFYNLGSPVAAVFFNCQRETGSGGRTFR- : 177
PsFTa2      : QQC--RQIV--YAPGWRQNF-NTREFVELYNLELPVAAVFFNCQREAGSGGRTFR- : 176
CaFTa2      : QQC--QQVV--FAPGWRQNF-NTREFAELYDLELPVAAVFFNCQRETGSGGRTFR- : 176
GmFTa1/2a : QQF--RQRV--YAPGWRQNF-NTREFAELYNLGLPVAAVFFNCQRESGSGGRTF-- : 175
GmFTa1/2b : QQF--RQRV--YAPGWRQNF-NTREFAELYNLGLPVAAVFFNCQRETGSGGRTF-- : 175
GmFT-like : MKS--GRIV--KAPEKRTNF-NTTEFAAKYEL-QDVAGVFFNSRRRG--------- : 163
MtFTa3      : QLG--RETV--YAPGWRQNF-NTREFAELYNLGLPVAAAYFNIQREHGSGGRRL-- : 175
CaFTa3      : QLG--RETV--YAPGWRQNF-NTREFAELYNLGLPVSSVYYNIQREAGSGGRRLC- : 178
GmFTa3a     : QLG--RETV--YAPGWRQNF-NTKEFAELYNLGLPVAAVYFNIQRESGSGGRRLY- : 176
GmFTa3b     : QQG--RETV--YAPGWRQNF-ITREFAELYNLGLPVAAVYFNIQRESGCGGRRLC- : 176
GmFTa3c     : QQG--RETV--YAPGWRQNF-ITREFAELYNLGLPVAAVYFNIQRESGCGGRRL-- : 175
GmFTa3d     : QLG--RETV--YAPGWRQNF-NTREFAELYNLGLPVAAVYFNIQRESGSGGRRLYH :  64
MtFTc       : QLG--RDTV--FAPDWRHNF-NTTNFAEINNLV-IVASVYFNCQRERGCGGRRC-- : 171
PsFTc       : QLG--RDTV--FAPEWRHNF-NTRNFAEINNLV-IVGSVYFNCQRERGCGGRRC-- : 174
CaFTc       : QLG--RDTV--FAPEWRHNF-KTRNFAEINNLV-IVASVYFNCQRERGCGGRRS-- : 176
GmFTc1      : QLG--RDTV--ITPEWRHNF-NSRNFAEINNLA-PVAAAYANCQRERGCGGRRY-- : 171
MtFTb1      : QPC--RHTV--LAPGWRQNF-ITRDFAEFYNLGLPVAALYFNCQRENGSGGRRLII : 173
PsFTb1      : QPC--RHTI--LPPGWRQNF-IIRDFAEIYNLGSPVAALYFNCQRQNGSGGRRMII : 178
MtFTb2      : QPC--RHTV--LAPGWRQNF-ITRDFAEFYNLGLPVAALYFNCQRENGSGGRRMVI : 178
PsFTb2      : QPC--RHTI--LPPGWRQNF-ITRDFAEVYNLGSPVAALYFNCQRENGSGGRRMIT : 178
CaFTb       : QPC--RHTI--LAPGWRQNF-VTRDFAEVYNLGLPVAALYFNVQRETGSGGRRMII : 179
GmFTb1      : QPR--RMSI--PAPGWRQNF-ITRDFAEYYNLGLPVAAVYFNCQRQGGSGGRRLML : 177
GmFTb2      : -----ENVF--TSSGWRQNYIMTRDFA--YNLGLPVAAVYFNCQRQGGSGERRLML :  86
GmFTb3      : QLR--RLTL--QPPGWRQNF-NTRDFAEIYNLGLPVAAMYFNCKRENDQSSGRRR- : 176
GmFTb4      : QSG--RQTI--YAPGWRQNF-NTRDFSEVYNLGLPVAATYFNCKRQNNSARDGRRT : 173
GmFTb5      : QFD--RQIV--HAPRWRQNF-NTRDFAEVYNLGSPVAAVYFNCQREGGWGGRRR-- : 175

AtCEN       : QTR--RGSV-VSVPSYRDQF-NTREFAHENDLGLPVAAVFFNCQRETAARRR---- : 175
AtTFL1      : QKQ--RRVI-FPNIPSRDHF-NTRKFAVEYDLGLPVAAVFFNAQRETAARKR---- : 177
MtTFL1a     : QKN--RESV-TASPSSRDYF-NTRNFASQNDLGLPVAAVYFNAQRETAARRR---- : 174
PsTFL1a     : QRA--RDSV-RATPSSRDHF-NTRSFASQNDLGLPVAAVYFNAQRETAARRR---- : 174
CaTFL1a     : QKS--RESV-MTTPSSRDHF-NTRNFASQNDLGLPVAAVYFNAQRETAARRR---- : 174
GmTFL1a1    : QKR--RQCV--TPPTSRDHF-NTRKFAAENDLALPVAAVYFNAQRETAARRR---- : 173
GmTFL1a2    : QKR--RQCV--TPPTSRDHF-NTRKFAAENDLGLPVAAVYFNAQRETAARRR---- : 173
MtTFL1b     : QKR--RQTV-MKIPTSRDLF-NTKKFAQDNDLGPPVAAVFFNAQRETAARRR---- : 173
PsTFL1b     : QKR--RQTV-MKIPTSRDLF-NTQKFAQDNDLGPPVAAVFFNAQRETAARRR---- : 173
CaTFL1b     : QKR--RQTV-MKIPTSRDHF-NTKKFAEDNDLGPPVAAVFFNAQRETAARRR---- : 177
GmTFL1b1    : QKR--RQGV-LKTPTTRDLF-NSRSFAEENELGPPVAAVFFNAQRETAARRR---- : 156
GmTFL1b2    : QKR--RGSD--ENSNNKX------------------------------------ : 140
MtTFL1c     : QEKGKKHSI--VAPFSRDHF-NTRAFSAQNDLGVPVAAAYFNARRATAPRRRAS-- : 177
PsTFL1c     : QKRGNKYSI--TCPFSRDHF-NTRNFADQNDLGVPVAAAYFNARRATAPRRR---- : 173
CaTFL1c1    : QKK--KHSI--TTPSSRDHF-NTRSFSMQNDLGVPVAAAYFNARRPTAGRKPTYI- : 176
CaTFL1c2    : QKK--KHSI--TTPSSRDHF-NTRSFSMQNDLGVPVAAAYFNARRPTAARKPTYI- : 176
CaTFL1c3    : QKK--KHSI--TTPSSRDHF-NTRSFSMQNDLGVPVAAAYFNARRPTAGRKPTYI- : 127
GmTFL1c1    : QKR--RQCV-TRPPSSRDHF-NTRKFSAENDLGLPVAAVYFNAQRETAARRR---- : 174
GmTFL1c2    : QKR--RQCV-TRPPSSRDHF-STRKFSAENDLGLPVASVYFNAQRETAARRR---- : 173
```

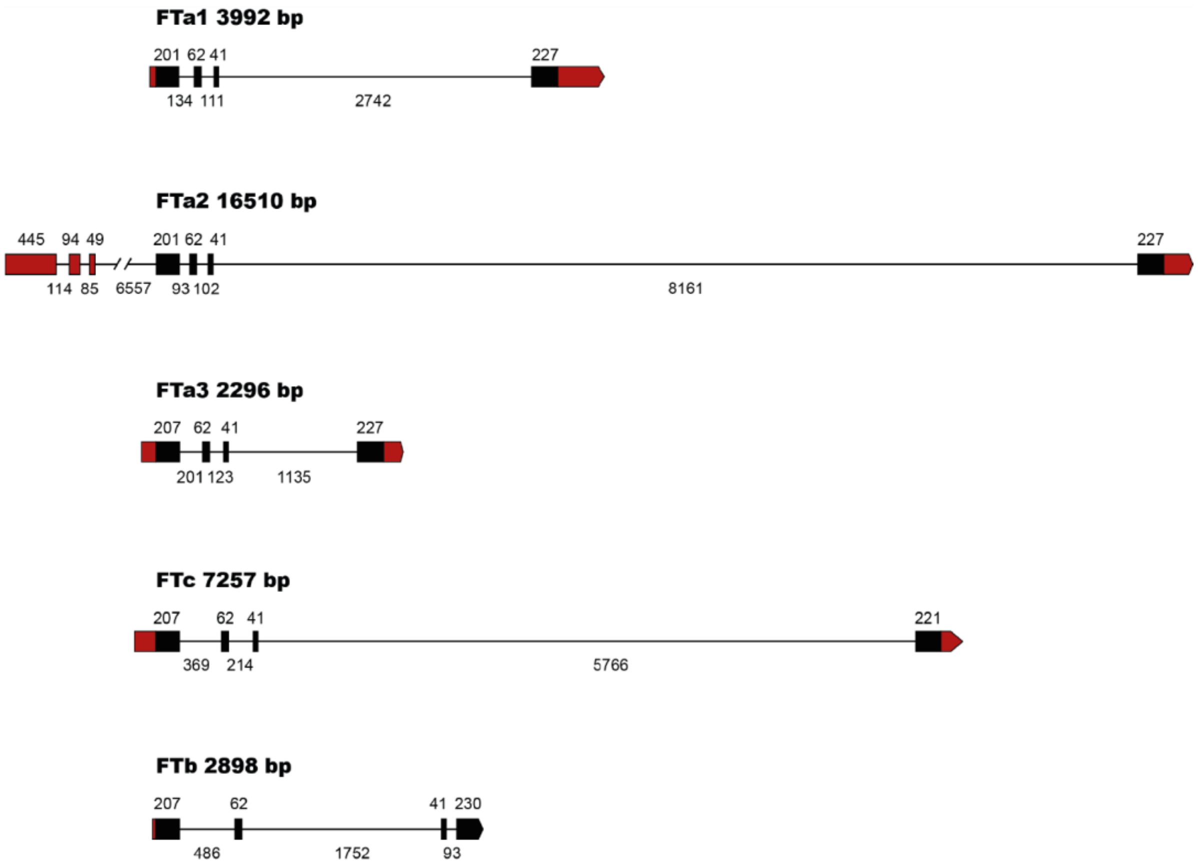

Supplementary Figure 10. Graphic representation of the genomic sequences of the five chickpea *FT* genes. Black boxes indicate exons (sizes over the line, in bp), red boxes the 5' and 3' untranslated regions and lines correspond to introns (sizes under the line).

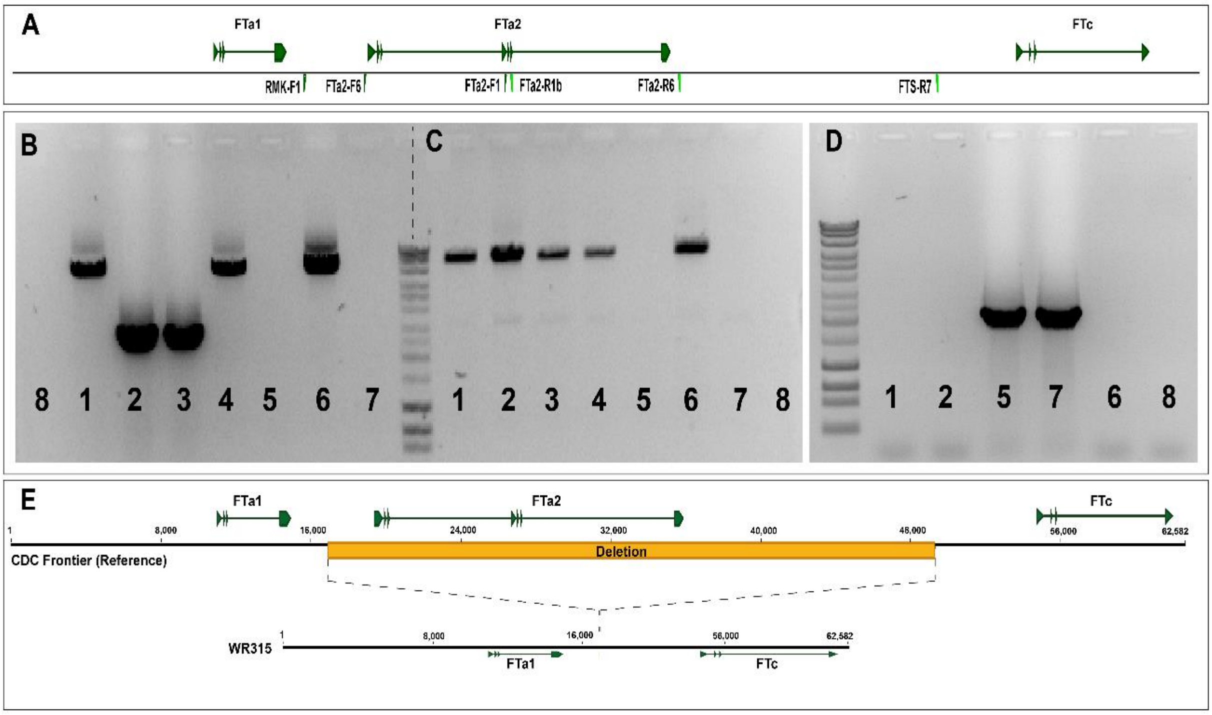

Supplementary Figure 11. (**A**) Schematic diagram of the region of chromosome 3 containing the *FTa1-FTa2-FTc* cluster. The black line represents genomic DNA sequence with *FTa1*, *FTa2* and *FTc* gene models shown at top in green (triangles representing exons and line corresponding to introns). The position of the primers used are indicated below; forward and reverse primers are presented in dark and light green, respectively. The failure to amplify any region of *FTa2* in some lines, shown by the electrophoresis gel of the products obtained with primers FTa2-F6/FTa2-R1b (**B**) and FTa2-F1/FTa2-R6 (**C**), suggested a deletion of the gene. 7 chickpea accessions were tested, as follows: 1-ICC4958; 2-PI489777; 3-Cr5-9; 4-CA2156; 5-WR315; 6-ILC3279; 7- ICCV2; 8-SDW. To determine the extent of the deletion, several primers were tested in the *FTa1-FTa2* and *FTa2-FTc* intergenic regions. The primer pair RMK-F1/FTS-R7 successfully yielded a band (**D**). Since the expected size of the amplicon in (**D**) is 33.7 kb according to CDC Frontier genome used as reference, the presence of a 2-3 kb band indicated a probable deletion spanning ~ 30kb. Sequencing of the band confirmed the deletion of 32kb that completely eliminates *FTa2* and includes part of the *FTa1-FTa2* and *FTa2-FTc* intergenic regions (**E**). All PCRs were performed in a final volume of 25 µL containing 50 ng template DNA, 5 µL of 5x reaction buffer, 10 mM dNTPs, 0.2 µM of each primer, 50 mM MgCl2, 0.1 µL of MangoTaqTM DNA polymerase (Bioline, Australia) and autoclaved Milli-Q water to final volume. Reactions were performed in a thermal cycler using the following program: an initial denaturation of 5 min at 94°C, followed by 35-40 cycles (94°C for 40 seconds, 58oC annealing temperature and 10 min extension) and a final extension of 10 minutes at 72°C. Primers sequences: FTa2-F6 AAGCCCACAACCCACCTAAGGG; FTa2-R1b ACTAGCCCCAGCAGTTGAAG; FTa2-F1 TAGGCGGAAACGATCTCA GG; FTa2-R6 GCCATAAACCTCTGTCGAACGGC, RMK-F1 ACTGTTCTGCACACAGTGGCTACC; FTS-R7 AGGCCAAAGACAAGATCCCG.

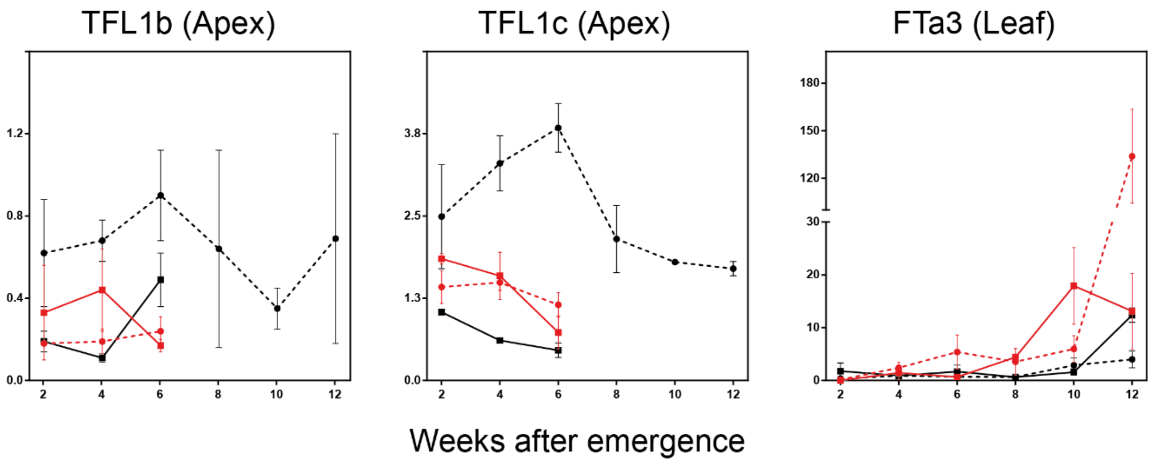

Supplementary Figure 12. Relative expression of flowering-related chickpea genes in CRIL2 parental lines. ICC4958 and PI48977 are represented by red and black lines, respectively. Continuous lines/squares indicate long day condition while dashed lines/circles represent short day. TFL1b and TFL1c expression was measured only in apex and FTa3 expression was measured in both tissues but found only in leaf. The average ± SE of 2 biological and 2 technical replicates is shown, and transcripts were normalized against ACTIN.

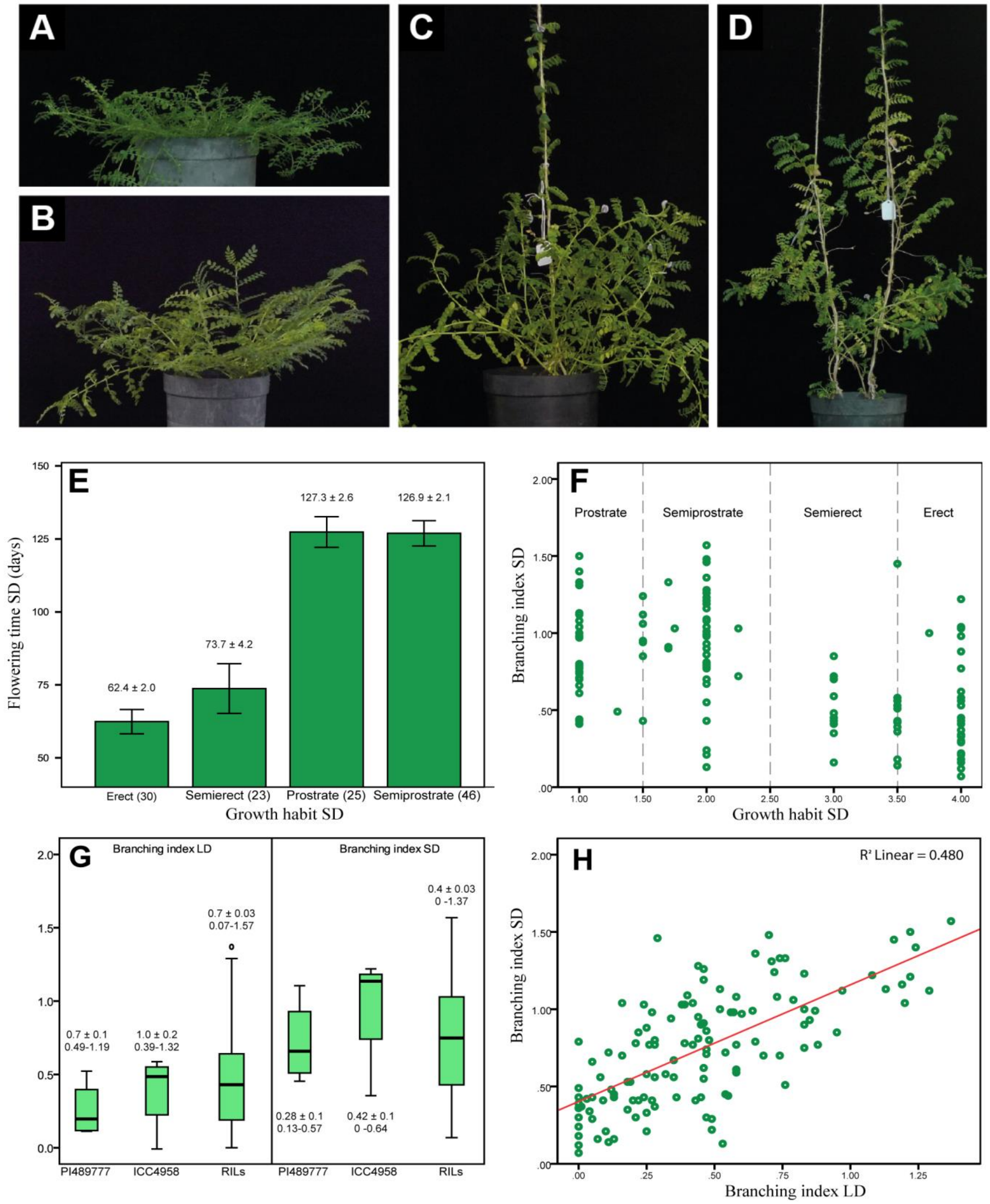

Supplementary Figure 13. Variation in growth habit (GH) observed in the CRIL2 population, illustrated by representative examples of the four categories used in the present study: Prostrate (**A**), semi-prostrate (**B**), semi-erect (**C**) and erect (**D**). (**E**) Relationship between GH and flowering phenotype (DTF) of the CRIL2 population grown under SD. Numbers over bars indicate mean DTF ± standard error and those between parentheses correspond to the number of RILs in each category. (**F**) Relationship between GH and branching index (BI) of the CRIL2 population grown under SD. Boxplot illustrating the variation in branching index displayed by CRIL2 parental lines and RILs under LD and SD (**G**). Box edges denote the lower and upper quantiles with median value in the middle of the box. (**H**) Relationship between BI of CRIL2 population grown under SD and LD photoperiod.

Supplementary Table 1. Details of the HRM markers developed in this study for each population, and identity (NCBI GeneID) of the genes targeted by them.

Marker	Population	Fw Sequence	Rv Sequence	GeneID
**LHY**	CRIL2/RIP12	AAACCAACTAAGCATACCCT	TGAGCATCACTCATTCACCA	101500635
	RIPs8/5	CGTCACACTTGTAATCTTCATTCC	AGTTTTCCCCCTTTAATAATGTGG	
**CP450**	CRIL2/RIP12	CACAAAATAGAGAACAATGACAGC	ACTTTTCCCTTTGCATGTAGG	101502624
	RIPs8/5	AGAGTTGTATAGTTGTTAAGGATG	GTGTGTGTGTTTATCAATTTAAGC	
**CDF2d**	CRIL2/RIP12	TGGTTCCAATTAAGTTTCAAGTG	AGTCAAGTGTTTGGTAAGAGTTG	101500722
	RIPs8/5	AGTCGATGCTTAATCTTCAACAGC	AGATCTGCATAAAGATGGTTCC	
**WUS11**	CRIL2/RIP12	CAGCCTGGTAATTAACTGCATC	ATGATTTTGAGCAATTATTCTGTG	101503157
	RIPs8/5	GCATAACCTAGAGTGATCGAGC	CTACTCTGACTTAATGGGTTCG	
**FTa1**	CRIL2/RIP12	AATCATCCCCAAGAGATCAA	TGCACAGTCATTGTGTTTCG	101497376
	RIPs8/5	ACTGTTCTGCACACAGTGGCTACC	AAAGAGATCTAACACATTTTGC	
			GAGGAACACGACGCCTCC	
**ING2**	CRIL2/RIP12	CATCTCATGGCTCTAAGAAAGG	ATTTCTTTAAGCAATTTCTCAATGG	101503708
**SUVH4**[1]	CRIL2/RIP12	TTCGCCGTCACTACCTCG	GGAGATTAAGCTTCGGAGG	101508428
**COLh**	CRIL2/RIP12	TGCTACATCATTCACCTAGTAACA	TGCCATGATATAGGAAGTCTTAGTT	101504031
**SOC1a**	CRIL2/RIP12	AAACAAAGAAAAACGAATGTGTCC	CGACATATAATTCATTGTGGACCG	101510775
**SCARECROW**[2]	CRIL2/RIP12	GAGACATGTTGTTGAACAGC	CTTGATGGTCCTCTAACAGC	101513767
**RAPTOR1**[2]	CRIL2/RIP12	CCCAATGCCATCCAAATCGG	CACACAACAACAATACCTAGGG	101514864
**ARF9**[2]	RIP12	GCAATATGGTGAGAAGAATTTTC	TGAGATAGGCAATTTAGTCCCTG	101491204
**ABI5**	CRIL2/RIP12	TTAGCACAAGAACGAGCGAGC	GTGAACGAAAAGGTGTTATGAAGG	101490892
**GATA9**[1]	CRIL2/RIP12	GAGGAATATGTCTTCTTCCATTCC	TGGAAAGAGTAATTTTCCCCCTA	101503040
**NAC100**[1]	CRIL2/RIP12	CAGGTCTTAGCAATGACACG	GCCCTATTTCTTCCCATGTC	101500623
**GIGANTEA**	CRIL2/RIP12	TGTTGAGTACTTGATTCAGTTTAC	TCAGTATATAGATGCATACCTCAGC	101511540
**ELF6**	CRIL2/RIP12	ACCACCCATTTCAGTTTGTTTAC	CCAGGGTCAAAATTATCATAGTCG	101509509
**TEMb**	CRIL2/RIP12	GAGTTGAGAAAATCTGTTTCAGCG	AGGTTCCGCGGAAAAGACG	101492303
**PHYA1**	CRIL2/RIP12	ACAATTGCCTTGTAATCGCC	GCCCAAGATATAACTGCTCAG	101506511
**CDF3b**	RIPs8/5	AACAACCGAAGAAAAATAGG	GAATTTGTATAATGTTTATCTTCG	101499964
**COLg**	RIPs8/5	AGAGACTCTGAAGGTGTCCC	CAGTGGCTCGGAGAAAGTGG	101499146
**AP2-like**	RIPs8/5	AACAAACATCGTCACATCACC	CCTTGTGCTATTTAGTGTTCTGC	101502947
**LOB189**	RIPs8/5	ACAAAATCAATACAAGCAAACC	TGCAACCGTTAGTTTGTTTGG	101508422
**PRSP**	RIPs8/5	GATACATTTTCGCTCAAACTATG	TGCGTTGAAAAGTGTTTTATTAGC	101491522
**PRT6**	RIPs8/5	AAATTTTCATTCTCCTAAGACAGT	ACGGTCCAACCCAACGTATA	101506928
**WRKY**	RIPs8/5	TTCTGAGAGCACCGTGATGG	AGCATCTCCAACTGTAATTAAATG	101511519

(1) Primers designed by Saxena et al (2014)
(2) Primers designed for this study based on SNPs described by Saxena et al (2014)

Supplementary Table 2. Sequence and product size of the primers used to measure the expression of flowering related genes in chickpea

Gene	Fw primer	Rv primer	Product size (bp) Genomic	cDNA	GeneID
ACTIN	ATTGTCTTGAGTGGTGGTTCT	TTCCTCTCTGGTGGTGCTAC	(Verma et al. 2013)		
PIM	GAACTTCAGAGTCTGGAACAGC	CATTGTGCCTGTTGTTGAGC	472	188	101488241
UNI	TGCAACGCGTAACAGTGAACG	ACGAACAATGCCGTGAGTTCTTG	660	169	101503680
FTa1	TTGCCAATCAACCCAGAGCG	AGTGGGGTTACTTGGGCTAGG	236	101	101497376
FTa2	GTTCTGACGGTGGTTCTCTC	CGGAGGTTCACAAAAGAAGG	297	183	101496618
FTc	TGTTGGTGGTGAAGATCTAAGG	ATTCCTGCTGAAGGATTCG	6616	186	101508200
FTb	GGTGAGCTCAAACCCTCCCA	TCCCTCATATTTGGGTCACTAGG	618	131	101505276
FTa3	CCATCCCGGAGCATACACAGTC	TGCACCAAGCCCTAGCAATCC	1460	196	101515383
E1	AACGACAACAACAAGGGATCGG	TGCAGCCAACAAGAGTCTGC	120	120	101497661
TFL1b	TATACCGGGCACAACAGATG	GGAGGTCCAAGGTCATTGTC	584	195	101508699
TFL1c	ACGTTCCTGGCCCAAGTGAT	TGGATCCCTATGTTAGGCTTTGGT	1134	136	101491943

Supplementary Table 3. Description, physical position in the chromosome and GeneID of the 59 genes annotated between markers SUVH4 and GATA9, according to the genome assembly of cultivar CDC Frontier (ASM33114 v1; Varshney et al., 2013), available at the National Center for Biotechnology Information (NCBI; https://www.ncbi.nlm.nih.gov).

Marker	Position	GeneID	Description
SUVH4	25748486	101508428	histone-lysine N-methyltransferase, H3 lysine-9 specific SUVH4
	25765688	101509380	CLAVATA3/ESR (CLE)-related protein 4A-2-like
	25787922	101509702	uncharacterized protein LOC101509702
	25849917	101510346	protein Mpv17-like, partial
	25899924	101510883	N-alpha-acetyltransferase MAK3
	25908342	101511521	cytochrome b-c1 complex subunit Rieske-4, mitochondrial-like
	25911181	101511199	uncharacterized protein LOC101511199
	25920520	101511847	uncharacterized protein LOC101511847
	25929005	101512385	uncharacterized protein LOC101512385
	25945170	101512699	LRR receptor-like serine/threonine-protein kinase RPK2
	25987693	101513246	UPF0496 protein At3g19330-like
	26006715	101513575	putative serine/threonine-protein kinase isoform X2
	26029555	101514543	chalcone synthase
	26038727	101505971	uncharacterized protein LOC101505971
	26049105	101514869	jasmonate O-methyltransferase-like isoform X2
	26056836	101506278	jasmonate O-methyltransferase-like
	26073603	101506600	3,7-dimethylxanthine N-methyltransferase-like
	26105741	101515408	7-methylxanthosine synthase 1-like
	26119620	101506937	ethylene-responsive transcription factor LEP
	26135351	101515744	nudix hydrolase 12, mitochondrial
	26162323	101488713	calcium-binding mitochondrial carrier protein SCaMC-1-like
	26169052	101489051	uncharacterized protein LOC101489051
	26187333	101489383	histidine biosynthesis bifunctional protein hisIE, chloroplastic
	26191225	101489915	uncharacterized protein LOC101489915
	26198363	105851804	glycine-rich cell wall structural protein 1-like
	26211557	101490249	syntaxin-22-like
	26221867	101490568	syntaxin-22-like
	26223872	101490780	uncharacterized protein LOC101490780
	26228217	101491110	DNA cross-link repair protein SNM1 isoform X1
	26233285	101491643	uncharacterized protein LOC101491643
	26237155	101491949	uncharacterized protein LOC101491949
	26259086	101492622	uncharacterized protein LOC101492622
	26268535	101492953	probable xyloglucan endotransglucosylase/hydrolase protein B
	26275124	101493288	probable xyloglucan endotransglucosylase/hydrolase-like precursor
	26293522	101493610	glyceraldehyde-3-phosphate dehydrogenase A, chloroplastic

Supplementary Table 3. Continued

	26300947	101493929	pentatricopeptide repeat-containing protein At3g26630, chloroplastic
	26304938	101494250	WD repeat-containing protein LWD1
	26309206	105851806	pentatricopeptide repeat-containing protein At3g26630, chloroplastic-like
	26315891	101494555	acetyl-coenzyme A synthetase, chloroplastic/glyoxysomal
	26325185	101494873	eukaryotic peptide chain release factor subunit 1-3
	26331419	101495197	importin-13 isoform X2
	26353855	101495954	INO80 complex subunit D-like
	26365580	101496291	omega-hydroxypalmitate O-feruloyl transferase
FTa1	26393854	101497376	protein FLOWERING LOCUS T-like
	26409508	101496618	protein HEADING DATE 3A-like isoform X1
	26437711	101508200	protein FLOWERING LOCUS T-like
	26446273	101497706	transmembrane and coiled-coil domain-containing protein 1-like
	26454060	101498244	aquaporin SIP1-2-like
	26459034	101498578	apyrase 2-like
	26465157	101499141	pentatricopeptide repeat-containing protein At5g15010, mitochondrial
	26476855	101499461	nucleoside-triphosphatase-like
	26505866	101499961	NAC transcription factor 29
	26513981	101500308	uncharacterized protein LOC101500308
NAC100	26542626	101500623	NAC domain-containing protein 100-like
	26551883	101500931	U11/U12 small nuclear ribonucleoprotein 65 kDa protein
	26560819	101501672	thioredoxin-like 1-1, chloroplastic
	26568175	101501985	CCR4-NOT transcription complex subunit 3 isoform X1
	26586013	101503370	glucan endo-1,3-beta-glucosidase 8-like
GATA9	26593826	101503040	GATA transcription factor 9-like

Supplementary Table 4. Accession number of the PEBP genes from five plant species; *Arabidopsis thaliana*, *Medicago truncatula*, *Pisum sativum* (Pea), *Glycine max* (soybean) and *Cicer arietinum* (chickpea). Soybean accessions were obtained from Wang et al. (2015) and the protein sequences retrieved from the Soybean Knowledge Database [http://soykb.org; Joshi et al. (2012)]. Proteins sequences retrieved from the accessions listed were used in the alignment displayed in Supplementary Figure 10.

**Arabidopsis**		**Medicago**		**Pea**	**Chickpea**		**Soybean**	
**MFT**	AT1G18100		Medtr8g106840	PsCam040701		LOC101504081	**GmMFTa**	Glyma05g34030
							**GmMFTb**	Glyma08g05650
**BFT**	AT5G62040		Medtr0020s0120	PsCam044479		LOC101507903	**GmBFTa**	Glyma09g26550
							**GmBFTb**	Glyma16g32080
**ATC**	AT2G27550		-	-		-		-
**TFL1**	AT5G03840	**TFL1a**	Medtr7g104460	AY340579	**TFL1a**	LOC101506075	**GmTFL1b1**	Glyma12g30940
		**TFL1b**	Medtr2g086270	AY340580	**TFL1b**	LOC101508699	**GmTFL1b2**	Glyma13g39360
		**TFL1c**	Medtr1g060190	AY343326	**TFL1c1**	LOC101495644	**GmTFL1a1**	Glyma03g35250
					**TFL1c2**	LOC101491943	**GmTFL1a2**	Glyma19g37890
					**TFL1c3**	LOC101492277	**GmTFL1c1**	Glyma10g08340
							**GmTFL1c2**	Glyma13g22030
**FT**	AT1G65480	**FTa1**	Medtr7g084970	HQ538822	**FTa1**	LOC101497376	**GmFTc1**	Glyma19g28400
**TSF**	AT4G20370	**FTa2**	Medtr7g085020	HQ538821	**FTa2**	LOC101496618	**GmFTa3a**	Glyma16g26660
		**FTa3**	Medtr6g033040	-	**FTa3**	LOC101515383	**GmFTa3b**	Glyma16g04830
		**FTb1**	Medtr7g006630	HQ538824	**FTb**	LOC101505276	**GmFTa3c**	Glyma16g26690
		**FTb2**	Medtr7g006690	HQ538825			**GmFTa3d**	Glyma02g07650
		**FTc**	Medtr7g085040	HQ538826	**FTc**	LOC101508200	**GmFTa1/2a**	Glyma19g28390
							**GmFTa1/2b**	Glyma16g04840
							**GmFTb1**	Glyma08g47820
							**GmFTb2**	Glyma18g53670
							**GmFTb3**	Glyma18g53680
							**GmFTb4**	Glyma18g53690
							**GmFTb5**	Glyma08g47810
							**GmFT-like**	Glyma08g28470

Supplementary Table 5. Information about sunrise and sunset times (h = hours, m = minutes) in Cordoba, Spain (Latitude, Longitude: 37 52 51, - 4 46 44) and Jaen, Spain (Latitude, Longitude: 37 45 59, - 3 47 21) during the growing seasons of RIP12, RIP5 and RIP8. Source: Observatorio Astronómico Nacional, Instituto Geográfico Nacional, Ministerio de Fomento, Spain.

Cordoba, 2001										
Day	February		March		April		May		June	
	Sunrise	Sunset	Sunrise	Sunset	Sunrise	Sunset	Sunrise	Sunset	Sunrise	Sunset
	h m	h m	h m	h m	h m	h m	h m	h m	h m	h m
1	823	1843	751	1913	804	2042	723	2110	659	2136
2	823	1844	749	1914	803	2043	722	2111	658	2136
3	822	1845	748	1915	801	2044	721	2112	658	2137
4	821	1846	747	1916	800	2045	720	2112	658	2138
5	820	1847	745	1917	758	2046	719	2113	657	2138
6	819	1848	744	1918	757	2047	718	2114	657	2139
7	818	1849	742	1919	756	2048	717	2115	657	2139
8	817	1850	741	1920	754	2048	716	2116	657	2140
9	816	1851	739	1921	753	2049	715	2117	657	2140
10	815	1853	738	1921	751	2050	714	2118	656	2141
11	814	1854	736	1922	750	2051	713	2119	656	2141
12	812	1855	735	1923	748	2052	712	2120	656	2142
13	811	1856	733	1924	747	2053	711	2121	656	2142
14	810	1857	732	1925	745	2054	710	2121	656	2143
15	809	1858	730	1926	744	2055	709	2122	656	2143
16	808	1859	729	1927	743	2056	708	2123	656	2143
17	807	1900	727	1928	741	2057	707	2124	656	2144
18	805	1901	726	1929	740	2058	707	2125	657	2144
19	804	1902	724	1930	738	2059	706	2126	657	2144
20	803	1903	723	1931	737	2059	705	2127	657	2144
21	802	1904	721	1932	736	2100	704	2127	657	2145
22	800	1905	720	1933	734	2101	704	2128	657	2145
23	759	1906	718	1934	733	2102	703	2129	658	2145
24	758	1908	717	1935	732	2103	702	2130	658	2145
25	756	1909	815	2036	731	2104	702	2131	658	2145
26	755	1910	814	2037	729	2105	701	2131	658	2145
27	754	1911	812	2037	728	2106	701	2132	659	2145
28	752	1912	811	2038	727	2107	700	2133	659	2145
29			809	2039	726	2108	700	2134	700	2145
30			808	2040	724	2109	659	2134	700	2145
31			806	2041			659	2135		

Cordoba, 2004										
Day	February		March		April		May		June	
	Sunrise	Sunset	Sunrise	Sunset	Sunrise	Sunset	Sunrise	Sunset	Sunrise	Sunset
	h m	h m	h m	h m	h m	h m	h m	h m	h m	h m
1	824	1842	750	1913	804	2042	723	2110	658	2136
2	823	1843	749	1914	803	2043	722	2111	658	2136
3	822	1844	748	1915	801	2044	721	2112	658	2137
4	821	1845	746	1916	800	2045	720	2113	657	2138
5	820	1846	745	1917	758	2046	718	2114	657	2138
6	819	1847	743	1918	757	2047	717	2115	657	2139
7	819	1848	742	1919	755	2048	716	2115	657	2139
8	818	1849	740	1920	754	2049	715	2116	657	2140
9	816	1851	739	1921	752	2050	714	2117	656	2140
10	815	1852	737	1922	751	2051	713	2118	656	2141
11	814	1853	736	1923	749	2051	712	2119	656	2141
12	813	1854	734	1924	748	2052	711	2120	656	2142
13	812	1855	733	1925	746	2053	711	2121	656	2142
14	811	1856	731	1926	745	2054	710	2122	656	2143
15	810	1857	730	1927	744	2055	709	2123	656	2143
16	809	1858	728	1927	742	2056	708	2123	656	2143
17	807	1859	727	1928	741	2057	707	2124	656	2144
18	806	1900	725	1929	739	2058	706	2125	657	2144
19	805	1901	724	1930	738	2059	706	2126	657	2144
20	804	1903	722	1931	737	2100	705	2127	657	2145
21	803	1904	721	1932	735	2101	704	2128	657	2145
22	801	1905	719	1933	734	2102	704	2128	657	2145
23	800	1906	718	1934	733	2103	703	2129	658	2145
24	759	1907	716	1935	731	2103	702	2130	658	2145
25	757	1908	715	1936	730	2104	702	2131	658	2145
26	756	1909	713	1937	729	2105	701	2132	659	2145
27	755	1910	712	1938	728	2106	701	2132	659	2145
28	753	1911	810	2039	727	2107	700	2133	659	2145
29	752	1912	809	2040	725	2108	700	2134	700	2145
30			807	2040	724	2109	659	2134	700	2145
31			806	2041			659	2135		

Cordoba, 2014										
Day	February		March		April		May		June	
	Sunrise	Sunset	Sunrise	Sunset	Sunrise	Sunset	Sunrise	Sunset	Sunrise	Sunset
	h m	h m	h m	h m	h m	h m	h m	h m	h m	h m
1	823	1842	751	1912	805	2042	723	2110	659	2136
2	823	1843	750	1913	803	2043	722	2110	658	2136
3	822	1845	748	1914	802	2044	721	2111	658	2137
4	821	1846	747	1915	800	2045	720	2112	658	2137
5	820	1847	745	1916	759	2046	719	2113	657	2138
6	819	1848	744	1917	757	2046	718	2114	657	2139
7	818	1849	742	1918	756	2047	717	2115	657	2139
8	817	1850	741	1919	754	2048	716	2116	657	2140
9	816	1851	740	1920	753	2049	715	2117	657	2140
10	815	1852	738	1921	751	2050	714	2118	656	2141
11	814	1853	737	1922	750	2051	713	2119	656	2141
12	813	1855	735	1923	748	2052	712	2120	656	2142
13	811	1856	734	1924	747	2053	711	2120	656	2142
14	810	1857	732	1925	746	2054	710	2121	656	2143
15	809	1858	731	1926	744	2055	709	2122	656	2143
16	808	1859	729	1927	743	2056	708	2123	656	2143
17	807	1900	728	1928	741	2057	708	2124	656	2144
18	806	1901	726	1929	740	2058	707	2125	657	2144
19	804	1902	724	1930	739	2058	706	2126	657	2144
20	803	1903	723	1931	737	2059	705	2126	657	2144
21	802	1904	721	1932	736	2100	705	2127	657	2145
22	800	1905	720	1933	735	2101	704	2128	657	2145
23	759	1906	718	1934	733	2102	703	2129	658	2145
24	758	1907	717	1935	732	2103	703	2130	658	2145
25	756	1908	715	1935	731	2104	702	2130	658	2145
26	755	1909	714	1936	729	2105	701	2131	658	2145
27	754	1910	712	1937	728	2106	701	2132	659	2145
28	752	1911	711	1938	727	2107	700	2133	659	2145
29			709	1939	726	2108	700	2133	700	2145
30			808	2040	725	2109	659	2134	700	2145
31			806	2041			659	2135		

Cordoba, 2003										
Day	February		March		April		May		June	
	Sunrise	Sunset	Sunrise	Sunset	Sunrise	Sunset	Sunrise	Sunset	Sunrise	Sunset
	h m	h m	h m	h m	h m	h m	h m	h m	h m	h m
1	824	1842	752	1912	805	2042	724	2109	659	2135
2	823	1843	750	1913	804	2043	723	2110	658	2136
3	822	1844	749	1914	802	2043	722	2111	658	2137
4	821	1845	747	1915	801	2044	720	2112	658	2137
5	820	1846	746	1916	759	2045	719	2113	657	2138
6	819	1848	744	1917	758	2046	718	2114	657	2138
7	818	1849	743	1918	756	2047	717	2115	657	2139
8	817	1850	742	1919	755	2048	716	2116	657	2140
9	816	1851	740	1920	753	2049	715	2117	657	2140
10	815	1852	739	1921	752	2050	714	2117	656	2141
11	814	1853	737	1922	750	2051	713	2118	656	2141
12	813	1854	736	1923	749	2052	712	2119	656	2142
13	812	1855	734	1924	748	2053	711	2120	656	2142
14	811	1856	733	1925	746	2054	710	2121	656	2142
15	810	1857	731	1926	745	2054	709	2122	656	2143
16	808	1859	730	1927	743	2055	709	2123	656	2143
17	807	1900	728	1928	742	2056	708	2124	656	2144
18	806	1901	727	1929	740	2057	707	2124	656	2144
19	805	1902	725	1930	739	2058	706	2125	657	2144
20	803	1903	724	1931	738	2059	705	2126	657	2144
21	802	1904	722	1931	736	2100	705	2127	657	2145
22	801	1905	720	1932	735	2101	704	2128	657	2145
23	800	1906	719	1933	734	2102	703	2129	657	2145
24	758	1907	717	1934	732	2103	703	2129	658	2145
25	757	1908	716	1935	731	2104	702	2130	658	2145
26	756	1909	714	1936	730	2105	702	2131	658	2145
27	754	1910	713	1937	729	2106	701	2132	659	2145
28	753	1911	711	1938	727	2106	701	2132	659	2145
29			710	1939	726	2107	700	2133	659	2145
30			808	2040	725	2108	700	2134	700	2145
31			807	2041			659	2135		

Cordoba, 2008										
Day	February		March		April		May		June	
	Sunrise	Sunset	Sunrise	Sunset	Sunrise	Sunset	Sunrise	Sunset	Sunrise	Sunset
	h m	h m	h m	h m	h m	h m	h m	h m	h m	h m
1	824	1842	750	1913	804	2042	723	2110	658	2136
2	823	1843	749	1914	803	2043	722	2111	658	2137
3	822	1844	748	1915	801	2044	721	2112	658	2137
4	821	1845	746	1916	800	2045	720	2113	658	2138
5	820	1846	745	1917	758	2046	718	2114	657	2138
6	819	1847	743	1918	757	2047	717	2115	657	2139
7	818	1848	742	1919	755	2048	716	2116	657	2139
8	817	1850	740	1920	754	2049	715	2116	657	2140
9	816	1851	739	1921	752	2050	714	2117	657	2141
10	815	1852	737	1922	751	2051	713	2118	656	2141
11	814	1853	736	1923	749	2052	712	2119	656	2142
12	813	1854	734	1924	748	2052	711	2120	656	2142
13	812	1855	733	1925	746	2053	711	2121	656	2142
14	811	1856	731	1926	745	2054	710	2122	656	2143
15	810	1857	730	1927	744	2055	709	2123	656	2143
16	809	1858	728	1928	742	2056	708	2123	656	2143
17	807	1859	727	1928	741	2057	707	2124	656	2144
18	806	1900	725	1929	739	2058	706	2125	657	2144
19	805	1902	724	1930	738	2059	706	2126	657	2144
20	804	1903	722	1931	737	2100	705	2127	657	2145
21	802	1904	721	1932	735	2101	704	2128	657	2145
22	801	1905	719	1933	734	2102	704	2128	657	2145
23	800	1906	718	1934	733	2103	703	2129	658	2145
24	759	1907	716	1935	731	2104	702	2130	658	2145
25	757	1908	715	1936	730	2104	702	2131	658	2145
26	756	1909	713	1937	729	2105	701	2132	659	2145
27	755	1910	712	1938	728	2106	701	2132	659	2145
28	753	1911	710	1939	726	2107	700	2133	659	2145
29	752	1912	709	1940	725	2108	700	2134	700	2145
30			807	2041	724	2109	659	2134	700	2145
31			806	2041			659	2135		

Jaen, 2003										
Day	February		March		April		May		June	
	Sunrise	Sunset	Sunrise	Sunset	Sunrise	Sunset	Sunrise	Sunset	Sunrise	Sunset
	h m	h m	h m	h m	h m	h m	h m	h m	h m	h m
1	820	1838	747	1908	801	2038	720	2105	655	2131
2	819	1839	746	1909	800	2039	719	2106	655	2132
3	818	1840	745	1910	758	2039	718	2107	654	2132
4	817	1842	743	1911	757	2040	717	2108	654	2133
5	816	1843	742	1912	755	2041	716	2109	654	2134
6	815	1844	740	1913	754	2042	715	2110	654	2134
7	814	1845	739	1914	752	2043	713	2111	653	2135
8	813	1846	737	1915	751	2044	712	2111	653	2135
9	812	1847	736	1916	749	2045	711	2112	653	2136
10	811	1848	735	1917	748	2046	710	2113	653	2136
11	810	1849	733	1918	747	2047	709	2114	653	2137
12	809	1850	732	1919	745	2048	708	2115	653	2137
13	808	1852	730	1920	744	2049	708	2116	653	2138
14	807	1853	729	1921	742	2049	707	2117	653	2138
15	805	1854	727	1922	741	2050	706	2118	653	2139
16	804	1855	726	1923	739	2051	705	2119	653	2139
17	803	1856	724	1924	738	2052	704	2119	653	2139
18	802	1857	723	1925	737	2053	703	2120	653	2140
19	801	1858	721	1926	735	2054	703	2121	653	2140
20	759	1859	720	1927	734	2055	702	2122	653	2140
21	758	1900	718	1927	733	2056	701	2123	653	2140
22	757	1901	717	1928	731	2057	700	2124	654	2141
23	756	1902	715	1929	730	2058	700	2124	654	2141
24	754	1903	713	1930	729	2059	659	2125	654	2141
25	753	1904	712	1931	727	2100	659	2126	654	2141
26	752	1905	710	1932	726	2100	658	2127	655	2141
27	750	1906	709	1933	725	2101	657	2127	655	2141
28	749	1907	707	1934	724	2102	657	2128	655	2141
29			706	1935	722	2103	656	2129	656	2141
30			804	2036	721	2104	656	2130	656	2141
31			803	2037			656	2130		
